# Atomically Dispersed Fe‐Modified SnS for Sn‐Centered Peroxymonosulfate Activation Toward Acetaminophen Degradation

**DOI:** 10.1002/advs.78025

**Published:** 2026-09-27

**Authors:** Shuhui Liu, Yantao Wang, Ya Liu, Jingkai Lin, Shaobin Wang, Wenjie Tian

**Affiliations:** ^1^ School of Chemical Engineering Adelaide University Adelaide South Australia Australia

**Keywords:** atomic Fe‒S, electronic modulation, peroxymonosulfate, radicals, SnS nanobelt

## Abstract

SnS is a low‐cost and environmentally benign semiconductor for photocatalysis and piezocatalysis, but its direct use for peroxymonosulfate (PMS) activation remains limited. Here, we develop a facile Fe substitution strategy to construct atomically dispersed Fe‒S in SnS nanostructures (Fe‐SnS) for efficient PMS activation. The catalytic activity shows a volcano‐shaped dependence on Fe incorporation, with 5% Fe‐SnS (Fe/Sn molar ratio of 4.63%) giving the optimal performance, achieving efficient acetaminophen (APAP) removal with a rate constant of 1.23 min^‒1^, which is 3.84 and 176‐fold higher than those of SnS and commercial SnS, respectively. In situ X‐ray absorption spectroscopy reveals a site‐dependent activation mechanism in which Sn sites directly coordinate with O in PMS. Density functional theory calculations further show that Fe incorporation induces local electronic redistribution around neighboring Sn sites and enhances the Sn–PMS interfacial interaction. Fe incorporation also attenuates the negative surface charge of SnS, further facilitating PMS interaction. Mechanism studies revealed that APAP degradation proceeds via sulfate and hydroxyl radical‐dominated pathway. These findings clarify the distinct roles of host Sn and atomically dispersed Fe sites in PMS activation.

## Graphical abstract

Atomically dispersed Fe regulates the local electronic environment and surface properties of SnS, while PMS preferentially coordinates to Sn through Sn–O interaction. Fe incorporation further enhances the Sn–O_PMS_ interfacial interaction, facilitating Sn‐centered PMS activation and the generation of SO_4_^•−^/•OH for APAP degradation, thereby clarifying the distinct roles of host Sn and atomic Fe sites.

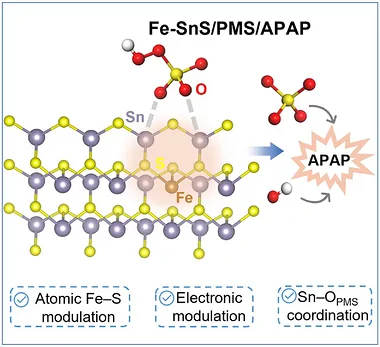

## 1 Introduction

Emerging contaminants, including pharmaceuticals and personal care products (PPCPs), per‐ and polyfluoroalkyl substances, endocrine‐disrupting compounds, polycyclic aromatic hydrocarbons, and brominated flame retardants, are increasingly detected in a wide range of environmental matrices [1, 2]. Among them, pharmaceuticals are frequently reported in wastewater and surface water systems, posing serious risks to ecosystems and human health due to their persistence and poor biodegradability [3]. Acetaminophen (APAP), a widely used analgesic and antipyretic, is often selected as a representative PPCP [4, 5], because of its high usage and continuous release into the environment. Owing to its chemical stability and high water solubility, conventional water treatment technologies are often ineffective in eliminating APAP from aquatic systems, leading to its continuous discharge into water bodies and associated environmental and public health concerns [6, 7]. With the increasing use of pharmaceuticals in the post‐pandemic era and an aging population, the demand for effective treatment technologies to address pharmaceutical‐laden wastewater has become increasingly urgent [8].

Among advanced oxidation processes (AOPs), peroxymonosulfate (PMS)‐based systems are widely used sulfate radical (SO_4_^•−^)‐related AOPs (SR‐AOPs) that generate SO_4_^•−^ and hydroxyl radicals (•OH) for the degradation of refractory organic pollutants [9, 10]. Homogeneous transition metal ions, e.g., Co^2+^, Fe^2+^, Cu^2+^, and Mn^2+^ can effectively activate PMS, but their practical application is limited by metal leaching and difficulty in recovery. These limitations have driven growing interest in heterogeneous catalysts with regulated active site structure and electronic properties for PMS activation, like transition metal oxides, carbon‐supported metal materials, etc [11, 12, 13, 14, 15]. Many heterogeneous SR‐AOPs often suffer from slow reaction kinetics, which increases oxidant consumption and operational cost [16, 17], highlighting the need for catalysts with well‐defined active sites and improved interfacial reactivity.

Metal chalcogenides have emerged as promising heterogeneous catalysts due to their tunable electronic structures and flexible lattice chemistry. Especially, tin monosulfide (SnS) is a group IV layered chalcogenide with a narrow bandgap (E_g_ ≈ 1.1–1.3 eV) and favorable electrical conductivity, which can facilitate interfacial electron transfer in catalytic reactions [18, 19, 20]. The electronic properties, together with low cost and relatively low toxicity, make SnS attractive for catalytic and environmental applications [21, 22, 23], and it has been studied in photocatalysis and piezocatalysis‐based water treatment [24, 25]. However, the exploration of SnS in AOPs systems remains limited, and the regulation of its surface reactivity and catalytic sites for PMS activation has not been systematically investigated. More importantly, the nature of the active sites in SnS and their specific catalytic roles during PMS activation remain poorly understood.

Currently, constructing well‐defined transition metal coordination sites has emerged as an effective strategy to enhance the catalytic performance of metal chalcogenides [26, 27]. Among various transition metals, iron (Fe) is particularly attractive because its variable valence states enable redox flexibility, which facilitates PMS activation [28, 29, 30]. Also, compared with other transition metals such as cobalt (Co), manganese (Mn), and copper (Cu), Fe is less toxic and more biocompatible [31]. For example, Fe doping in SnS_2_ has been reported to induce lattice distortion and enhance piezoelectric polarization, while Fe itself serves as a catalytic active center through valence‐state transformation in the subsequent self‐Fenton process [32]. Meanwhile, atomically dispersed Fe catalysts have attracted considerable attention for PMS activation because of their high atomic utilization and well‐defined local coordination environments. In many reported studies, atomically dispersed Fe sites have been identified as the primary active centers for PMS adsorption and activation, highlighting the direct catalytic function of Fe sites in different catalytic environments [33, 34]. However, the role of Fe incorporation in SnS, particularly the respective contributions of Fe and host Sn sites to PMS activation, remains insufficiently understood. When atomically dispersed Fe is introduced into SnS with intrinsic PMS activation capability, whether Fe becomes the primary PMS activation center and how the Fe sites and host Sn sites contribute to PMS activation remain unclear. Clarifying the distinct catalytic roles of these sites is essential for understanding the PMS activation mechanism of Fe‐modified SnS.

In this work, atomically dispersed Fe‒S sites were introduced to regulate the local structure and surface properties of SnS. At a low PMS dosage of 0.5 mM, the optimized Fe‐SnS samples exhibited markedly enhanced PMS activation for acetaminophen degradation, with activity showing a volcano‐shaped dependence on Fe incorporation over Fe/Sn molar ratios of 1.64 ‐ 8.87%. In situ XAS reveals distinct site‐dependent responses during PMS activation: Sn sites preferentially coordinate with O in PMS, while atomic Fe remains structurally persistent. Combined with density functional theory (DFT) calculations, these results indicate that Fe incorporation not only regulates the local electronic environment of neighboring Sn sites but also strengthens the Sn–O_PMS_ interaction, thereby facilitating Sn‐centered PMS activation. This work provides an atomic‐site regulation strategy for developing SnS‐based catalysts for PMS‐driven SR‐AOP water treatment and provides mechanistic insight into the distinct roles of host metal sites and atomically dispersed metal sites during PMS activation in metal chalcogenides.

## 2 Results and Discussion

### 2.1 Characterization

**FIGURE 1:**
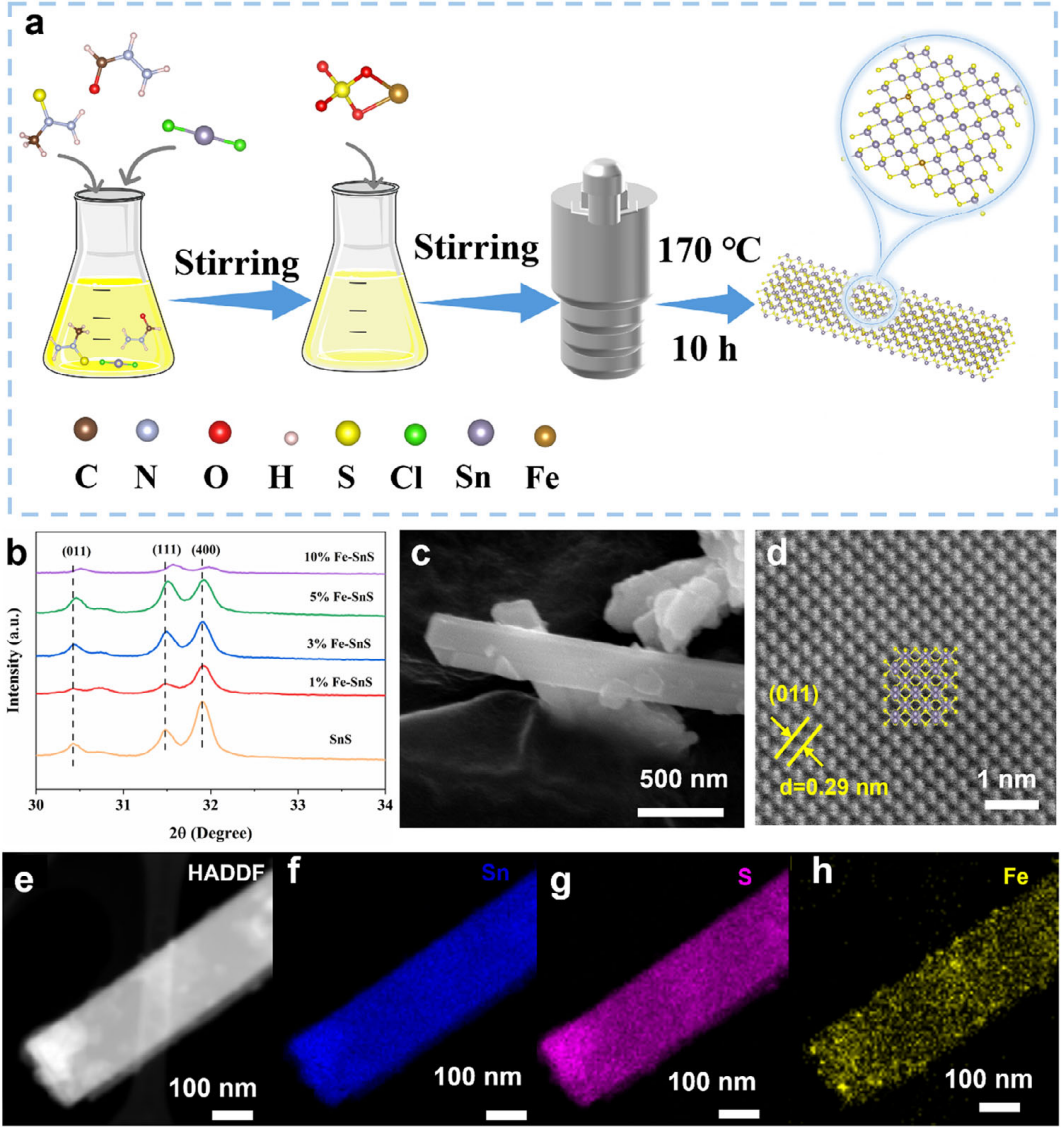
(a) Schematic synthesis process of SnS nanobelts with atomically dispersed Fe‐S sites. (b) XRD pattern of different ratios of Fe‐SnS, the enlarged view of (011), (111), and (400) crystal facets. (c) The SEM image of 5% Fe‐SnS. (d) Atomic‐resolution HAADF‐STEM image acquired from the nanobelt region of 5% Fe‐SnS corresponding to the (011) plane of SnS. (e–h) HAADF‐STEM elemental mapping images of 5% Fe‐SnS.

Fe‐SnS nanostructures were synthesized by introducing Fe precursor into the SnS nanobelt formation process via a modified one‐pot hydrothermal method [35], as illustrated in Figure 1a. A series of Fe‐SnS samples were prepared by varying the nominal Fe precursor ratio and denoted as 1% Fe‐SnS, 3% Fe‐SnS, 5% Fe‐SnS, and 10% Fe‐SnS, with actual Fe/Sn molar ratios of 1.64%, 3.89%, 4.63%, and 8.87%, respectively, based on inductively coupled plasma mass spectrometry (ICP‐MS) analysis (Table S1). As shown in X‐ray diffraction patterns (XRD, Figure S1), the diffraction peaks of SnS and Fe‐SnS samples are in good agreement with the orthorhombic structure of SnS (PDF #73‐1859), indicating that Fe incorporation does not change the main crystal phase of SnS. No additional peaks corresponding to crystalline Fe‐based phases were detected. For comparison, Fe_1‐x_S was synthesized under the same conditions without the use of Sn precursor and showed distinct diffraction peaks (Figure S2a). These reflections are absent in Fe‐SnS samples, even at high Fe loading (10% Fe‐SnS, Figure S2b). The enlarged XRD patterns (Figure 1b) show slight peak shifts of the (011), (111), and (400) reflections, with higher Fe precursor ratios (1%–5%). This can arise from the substitution of larger Sn^2+^ ions (∼0.93 Å) by smaller Fe^2+^ ions (∼0.61 Å), resulting in a slight lattice contraction. In contrast, 10% Fe‐SnS shows markedly broadened and weakened SnS diffraction peaks, indicating that excessive Fe incorporation disturbs SnS crystallization and decreases long‐range crystallographic ordering. Although no crystalline secondary phases were detected, the possible presence of amorphous Fe‐S species, surface disordered domains, or ultrasmall clusters below the XRD detection limit cannot be completely excluded. SEM images show that pristine SnS exhibits a nanobelt morphology, which is largely retained after Fe incorporation. Meanwhile, nanoparticles are formed on the surface of Fe‐SnS nanobelts, leading to a rougher surface texture (Figure 1c; Figures S3 and S4).

Representative atomic force microscopy (AFM) and piezoresponse force microscopy (PFM) measurements were conducted to compare the surface morphology and piezoelectric response of pristine SnS and 5% Fe‐SnS. As shown in Figures S5 and S6, pristine SnS exhibits a relatively smooth surface topography, whereas 5% Fe‐SnS displays a noticeably roughened surface with particulate features in the AFM topographic images, consistent with the SEM results. PFM measurements showed that pure SnS exhibited clear and typical amplitude‐voltage butterfly loops and well‐defined 180° phase change hysteresis loops (Figure S7a), while the piezoelectric response of 5% Fe‐SnS was significantly weakened, with no obvious butterfly loop or phase reversal (Figure S7b). These results suggest that Fe incorporation suppresses the piezoelectric response of SnS, making Fe‐SnS less suitable for piezoelectric‐driven catalysis compared with pristine SnS. High‐angle annular dark‐field scanning transmission electron microscopy (HAADF‐STEM) analysis (Figure 1d; Figure S8) was performed at different regions of 5% Fe‐SnS, and the nanobelt and nanoparticle regions show consistent lattice fringes with a d‐spacing of ∼0.29 nm, corresponding to the (011) plane of orthorhombic SnS. Examination of multiple regions did not reveal any Fe‐containing nanoparticles, clusters, or secondary phases, suggesting that Fe is highly dispersed in the SnS matrix. Because the HAADF‐STEM contrast is mainly dominated by the heavier Sn‐containing lattice columns, Fe atoms incorporated into the SnS framework cannot be distinguished as bright isolated spots, unlike single Fe atoms on light‐element carbon supports. These observations suggest that Fe is atomically dispersed within the SnS matrix. The corresponding energy‐dispersive X‐ray spectroscopy (EDS) elemental mapping shows well‐overlapped Sn and S signals across the nanobelt and surface nanoparticle regions, confirming their SnS nature, while the weak Fe signal generally follows the morphology/thickness contrast without obvious Fe‐rich aggregates (Figure 1e–h).

**FIGURE 2:**
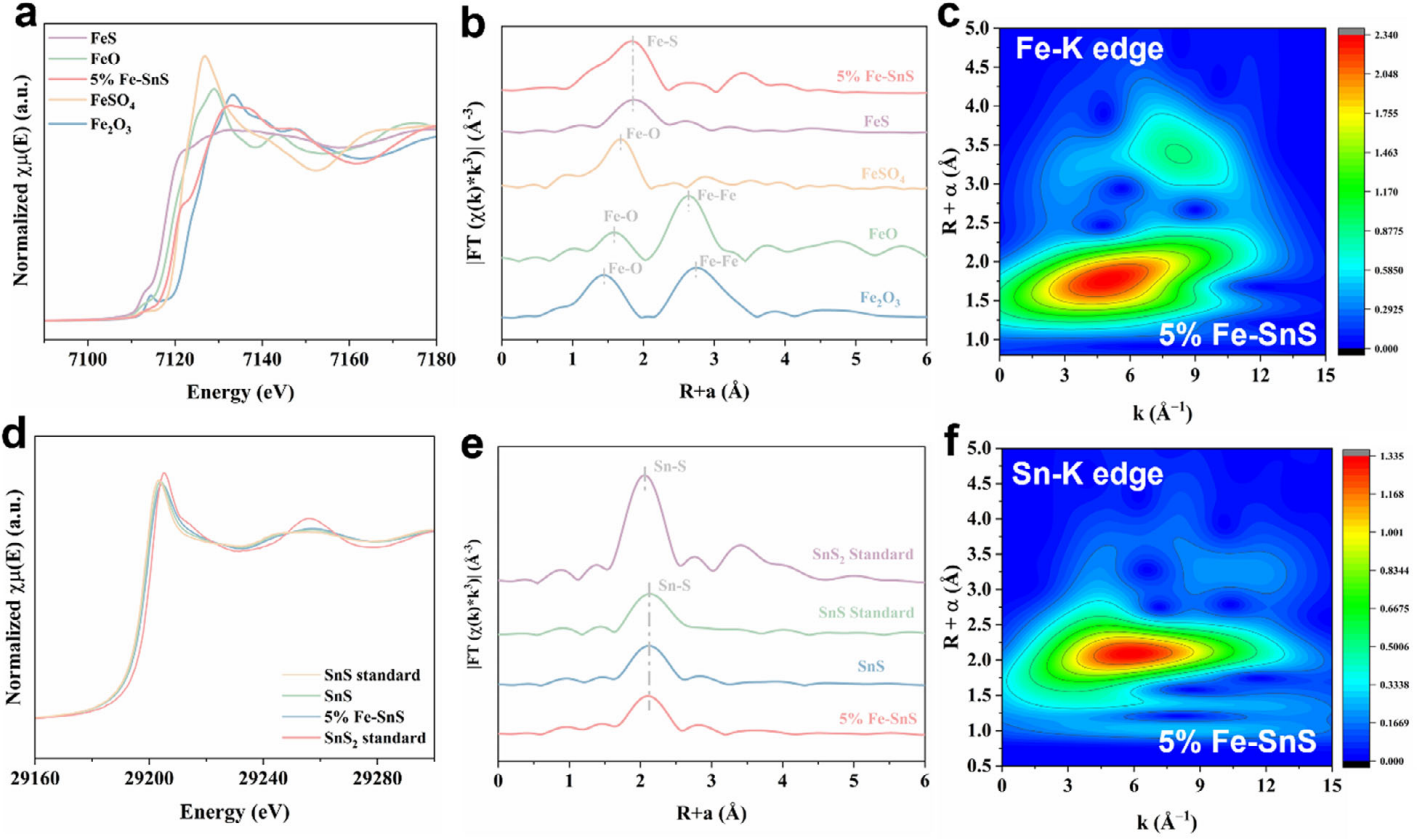
(a) XANES spectra of 5% Fe‐SnS and references at the Fe K‐edge. (b) FT k^3^‐weighted EXAFS spectra of 5% Fe‐SnS and references at Fe K‐edge. (c) WT‐EXAFS for Fe K‐edge for 5% Fe‐SnS. (d) XANES spectra of 5% Fe‐SnS and references at the Sn K‐edge. (e) FT k^3^‐weighted EXAFS spectra of 5% Fe‐SnS and references at Sn K‐edge. (f) Contour plots of Sn K‐edge WT‐EXAFS for 5% Fe‐SnS.

XAS analysis, including X‐ray absorption near‐edge structure (XANES), the Fourier transformed (FT) extended X‐ray absorption fine structure (EXAFS), and wavelet transform (WT) were performed to probe the oxidation state and coordination environment of Fe and Sn in 5% Fe‐SnS. The XANES spectrum at the Fe K‐edge (Figure 2a) revealed that the absorption edge of Fe atoms in 5% Fe‐SnS is very similar to that of FeO, suggesting that Fe is predominantly present in the +2 oxidation state [36]. In the Fourier‐transformed EXAFS (FT‐EXAFS) spectra (Figure 2b), the dominant peak for 5% Fe‐SnS is located at approximately 1.84 Å, closely corresponding to the Fe‒S coordination observed in FeS, which suggests that Fe atoms are primarily coordinated with S atoms [37]. The absence of Fe‒Fe scattering further confirms the lack of Fe aggregation and the atomic dispersion of Fe within the SnS framework. WT analysis (Figure 2c; Figure S9) further corroborates the Fe‐S coordination, with the dominant intensity in the WT contour of 5% Fe‐SnS located at similar R and k values to FeS, yet clearly distinct from those of FeO or FeSO_4_ [38]. Collectively, these results demonstrate that Fe atoms are atomically dispersed within the SnS framework, with Fe predominantly coordinated to S atoms to form Fe−S sites. Combined with the lattice contraction revealed by XRD, these observations are consistent with the possible substitutional incorporation of Fe at Sn sites. Sn K‐edge XAS further verifies the structural preservation of the SnS framework after Fe incorporation. The XANES spectrum of 5% Fe‐SnS closely resembles those of SnS and SnS standard, indicating that Sn mainly retains the +2 oxidation state (Figure 2d). The corresponding FT‐EXAFS spectrum shows a dominant first‐shell peak consistent with Sn‒S coordination, which is further supported by the Sn‒S scattering feature in the WT‐EXAFS contour (Figure 2e,f; Figures S10 and S11). XPS analysis also confirms that Sn predominantly exists as Sn^2+^ [19], with minor surface Sn^4+^ species [18], while the S 2p spectrum is consistent with lattice sulfide species in SnS (Figure S12).

### 2.2 Catalytic Performance of SnS Nanobelts With Atomically Dispersed Fe‒S Sites for PMS Activation

**FIGURE 3:**
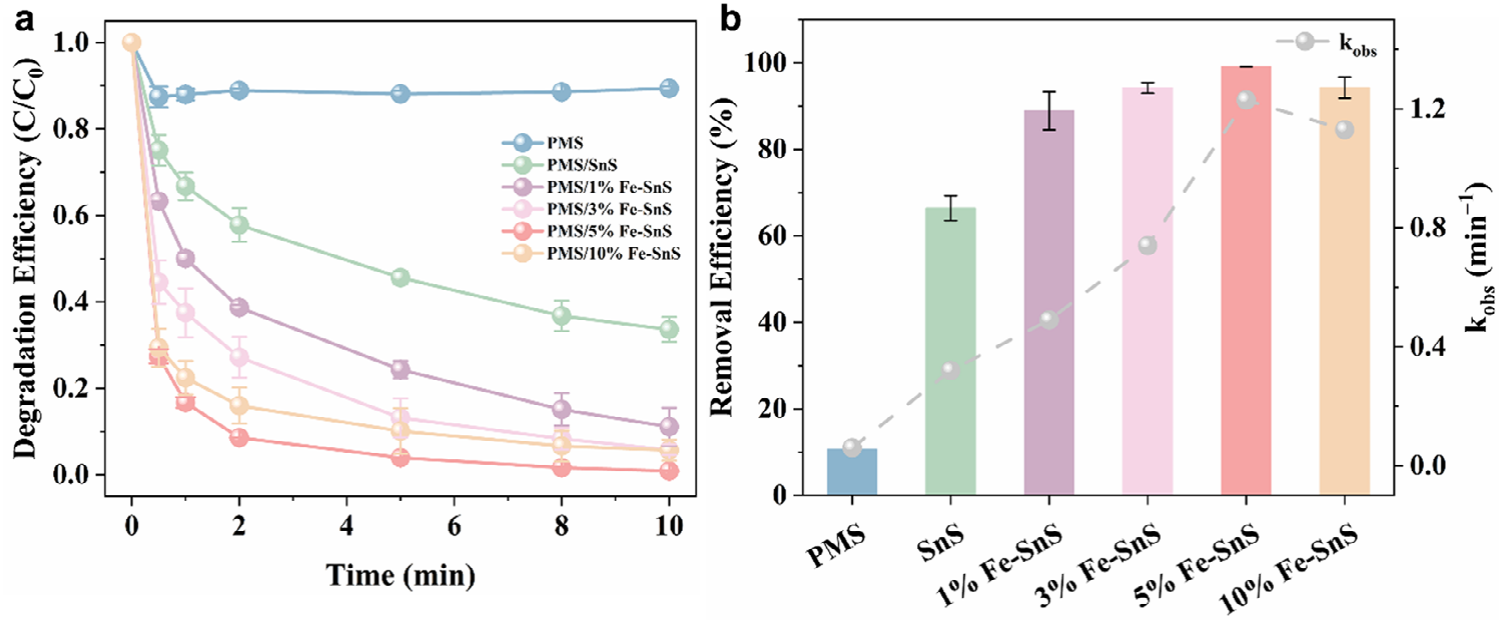
(a) Degradation rates of APAP with different reaction systems. (b) The corresponding reaction rate constants and removal rates in different reaction systems. Conditions: [Catalysts] = 0.4 g L^−1^, [PMS] = 0.5 mM, [APAP] = 20 µM, T = 25°C, pH = 5.84.

The catalytic performance of SnS and Fe‐SnS samples for PMS activation was evaluated by choosing APAP as a target contaminant. The catalyst dosage was optimized at a low PMS concentration of 0.5 mM (Figure S13), and the optimized conditions were used for subsequent experiments unless otherwise stated. Adsorption experiments showed that the adsorption effect of Fe‐SnS samples on APAP was negligible (Figure S14). Similarly, as shown in Figure 3a, the effect of PMS alone on APAP removal was minimal, with only ∼10% APAP removal. SnS/PMS system reached 66% APAP removal in 10 min, which exceeded commercial SnS (3%) and SnS_2_ (15%), highlighting the catalytic advantage of the as‐synthesized SnS (Figure S16). Fe‐SnS/PMS systems showed enhanced APAP degradation activity compared with SnS/PMS, displaying a volcano‐type dependence on the nominal Fe ratio. The optimized 5% Fe‐SnS/PMS system achieved near‐complete APAP removal within 10 min, while 1% Fe‐SnS and 3% Fe‐SnS showed moderate activity (Figure 3a). Further increasing the nominal Fe precursor ratio to 10% decreased the activity, likely due to disturbed SnS crystallinity. The degradation kinetics further show a volcano‐type dependence on the nominal Fe precursor. As shown in Figure 3b and Figure S15, 5% Fe‐SnS delivered the highest reaction rate constant (k_obs_) of 1.23 min^−1^, which is 3.84, 176, and 26 times higher than those of pristine SnS (0.32 min^−1^), commercial SnS (0.007 min^−1^), and SnS_2_ (0.047 min^−1^), respectively (Figure S16b,c). To further benchmark its catalytic performance, 5% Fe‐SnS was compared with recently reported APAP/PMS systems based on single‐atom catalysts, heteroatom‐doped carbons, transition‐metal oxides, sulfides and phosphides, as well as chlorine‐assisted activation (Table S3). Despite differences in catalyst dosage, solution pH, pollutant concentration, and other reaction conditions, 5% Fe‐SnS exhibits a notably apparent rate constant among the systems summarized in Table S3, at a relatively low PMS concentration of 0.5 mM. ICP analysis of the post‐reaction solution from 5% Fe‐SnS/PMS/APAP revealed Fe leaching of ∼308.51 µg L^−1^ (Table S2), which is very close to the drinking water regulatory limit of 0.3 mg L^−1^ [39, 40], while only minor Sn leaching (∼34.33 µg L^−1^) was detected. Given that dissolved Fe^2+^ may contribute to PMS activation, a control experiment was conducted using 5.52 µM Fe^2+^, corresponding to the measured Fe leaching concentration. Only a slight enhancement in APAP degradation was observed compared with PMS alone (Figure S17), indicating that the contribution of leached Fe^2+^ to homogeneous PMS activation was limited. Although no Fe_1‐x_S impurity phase was detected in 5% Fe‐SnS by structural characterizations, a control experiment was conducted to exclude the possible contribution of trace iron sulfide species to PMS activation. As shown in Figure S18, Fe_1‐x_S, dosed at an amount equivalent to the actual Fe content in 5% Fe‐SnS based on ICP analysis, showed negligible APAP degradation.

Beyond APAP, the 5% Fe‐SnS/PMS system showed applicability toward several representative emerging contaminants (Figure S19) and maintained effective APAP degradation over a wide initial pH range and in tap water (Figure S20). The effects of common coexisting anions and humic acid (HA) were also evaluated, with Cl^−^ and HA showing little influence, whereas HCO_3_^−^ and HPO_4_^2−^ caused noticeable inhibition (Figure S21). To further evaluate the environmental implications of APAP degradation, mineralization, transformation products, and their potential ecotoxicity were investigated. Although APAP was nearly completely removed within 10 min, the TOC showed no significant decrease, indicating limited mineralization (Figure S22). The liquid chromatography‐mass spectrometry (LC‐MS) and ECOSAR analyses further revealed the formation of major transformation products and an overall trend toward reduced predicted aquatic ecotoxicity (Figures S23 and S24, Tables S4 and S5). The reusability and structural stability of 5% Fe‐SnS were also evaluated. Although the catalytic activity decreased upon recycling, the post‐reaction XRD pattern remained similar to that of the fresh catalyst, whereas XPS analysis suggests partial surface oxidation of Sn^2+^ to Sn^4+^ after reaction (Figure S25), compared with that before reaction (Figure S12a).

### 2.3 Mechanistic and In Situ XAS Study of PMS Activation Over Fe‐SnS

**FIGURE 4:**
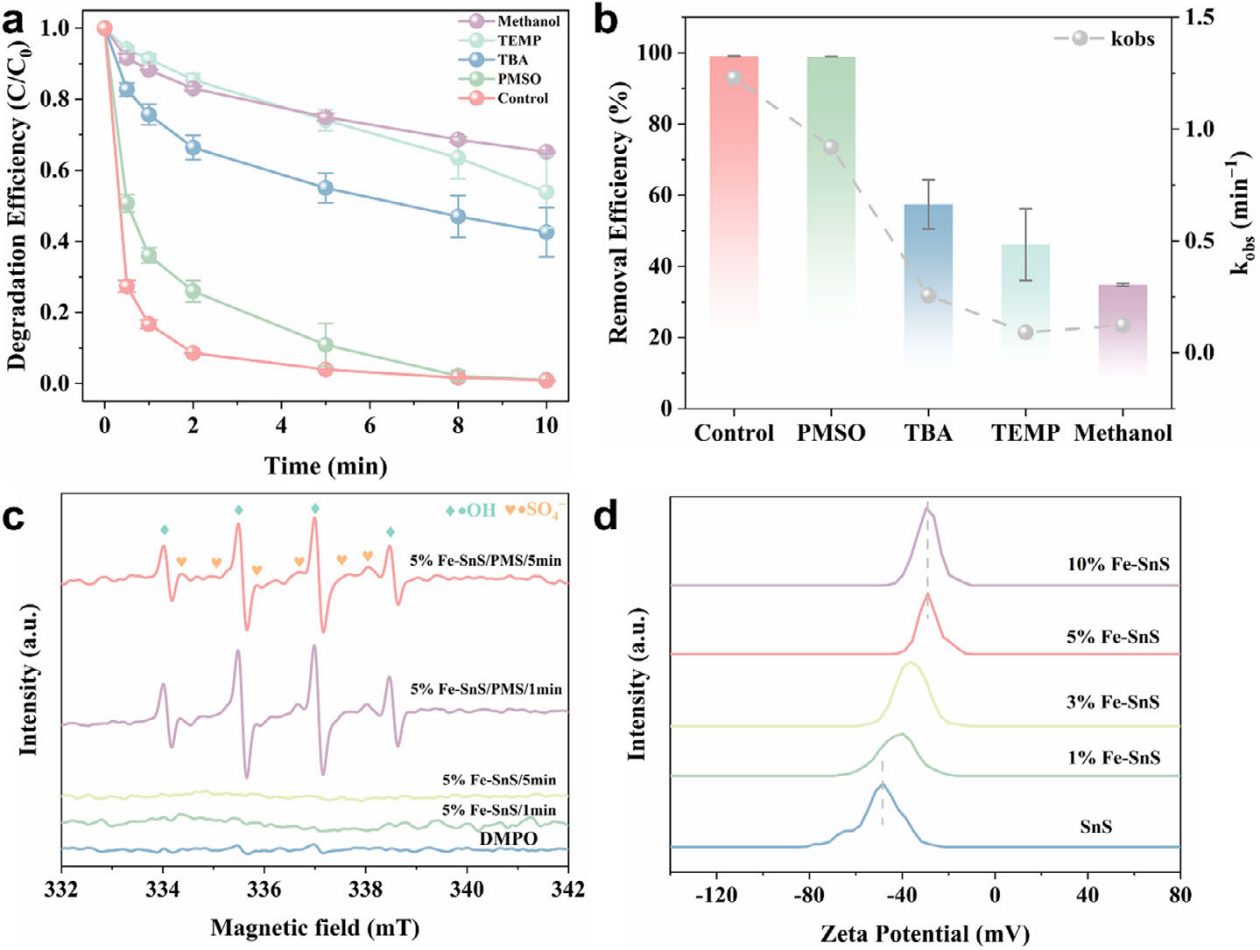
(a) Degradation of APAP in the presence of various scavengers in the 5% Fe‐SnS/PMS reaction system. (b) Degradation rate and apparent rate constant of APAP in the 5% Fe‐SnS/PMS catalytic system with different scavengers. (c) EPR spectra of •OH and SO_4_^•−^ in the 5% Fe‐SnS/PMS system by using DMPO as a trapping agent. (d) Zeta potential distribution profiles of SnS and Fe‐SnS samples. Experimental conditions: [PMS] = 0.5 mm, [Catalyst] = 0.4 g L^−1^, APAP = 20 µm, TBA = Methanol = 200 mm, [TEMP] = [PMSO] = 5 mm, T = 25°C.

To elucidate the underlying mechanism of PMS activation by 5% Fe‐SnS, a series of quenching experiments were performed using different scavengers. Tert‐butyl alcohol (TBA) was used as a scavenger for •OH, methanol for both •OH and SO_4_^•−^. While 2,2,6,6‐tetramethylpiperidine (TEMP) was used as a trapping reagent for singlet oxygen (^1^O_2_), and methyl phenyl sulfoxide (PMSO) for high‐valent metal species [41, 42]. PMSO probing showed negligible PMSO_2_ formation, suggesting that high‐valent iron species were not the dominant oxidants (Figure S26). Instead, the slight PMSO degradation was likely due to attacks by other reactive radical species [43, 44]. As shown in Figure 4a,b and Figure S27, upon TBA addition, k_obs_ decreased from 1.23 to 0.26 min^−1^, indicating that •OH was present in the 5% Fe‐SnS/PMS system. With methanol addition, the k_obs_ decreased to 0.12 min^−1^, confirming the coexistence of SO_4_^•−^ with •OH in the 5% Fe‐SnS/PMS system. Based on the k_obs_ values obtained from the scavenging experiments, the relative contributions of •OH and SO_4_^•−^ to APAP degradation were semi‐quantitatively estimated to be approximately 79% and 11%, respectively (Text S1), indicating that •OH was the predominant radical species in the reaction system. Moreover, electron spin resonance (ESR) measurements detected characteristic •OH and SO_4_^•−^ signals in the 5% Fe‐SnS/PMS system (Figure 4c). No ^1^O_2_ signal was observed (Figure S28). In parallel, electrochemical open‐circuit potential (OCP) measurements (Figure S29) and in situ Raman spectroscopy (Figure S30) ruled out the involvement of catalyst‐mediated electron transfer pathway in 5% Fe‐SnS/PMS/APAP system.

The effect of Fe incorporation on the surface charge properties of SnS was examined by Zeta potential measurements. As shown in Figure 4d and Figure S31, pristine SnS exhibits a negative Zeta potential of ‒48.5 mV. After Fe incorporation, the Zeta potential becomes gradually less negative, reaching −28.7 mV for 5% Fe‐SnS. Further increasing the Fe content to 10% does not lead to an additional decrease, with a comparable Zeta potential of −29.2 mV. This change can be attributed to the Fe‐induced redistribution of local surface charge in SnS. The formation of Fe‒S coordination and the substitution of Sn by Fe alter the electronic environment of neighboring Sn/S sites. Since PMS is negatively charged, the less negative zeta potential of Fe‐SnS indicates altered surface‐charge characteristics after Fe incorporation, which may influence its electrostatic interactions with PMS.

**FIGURE 5:**
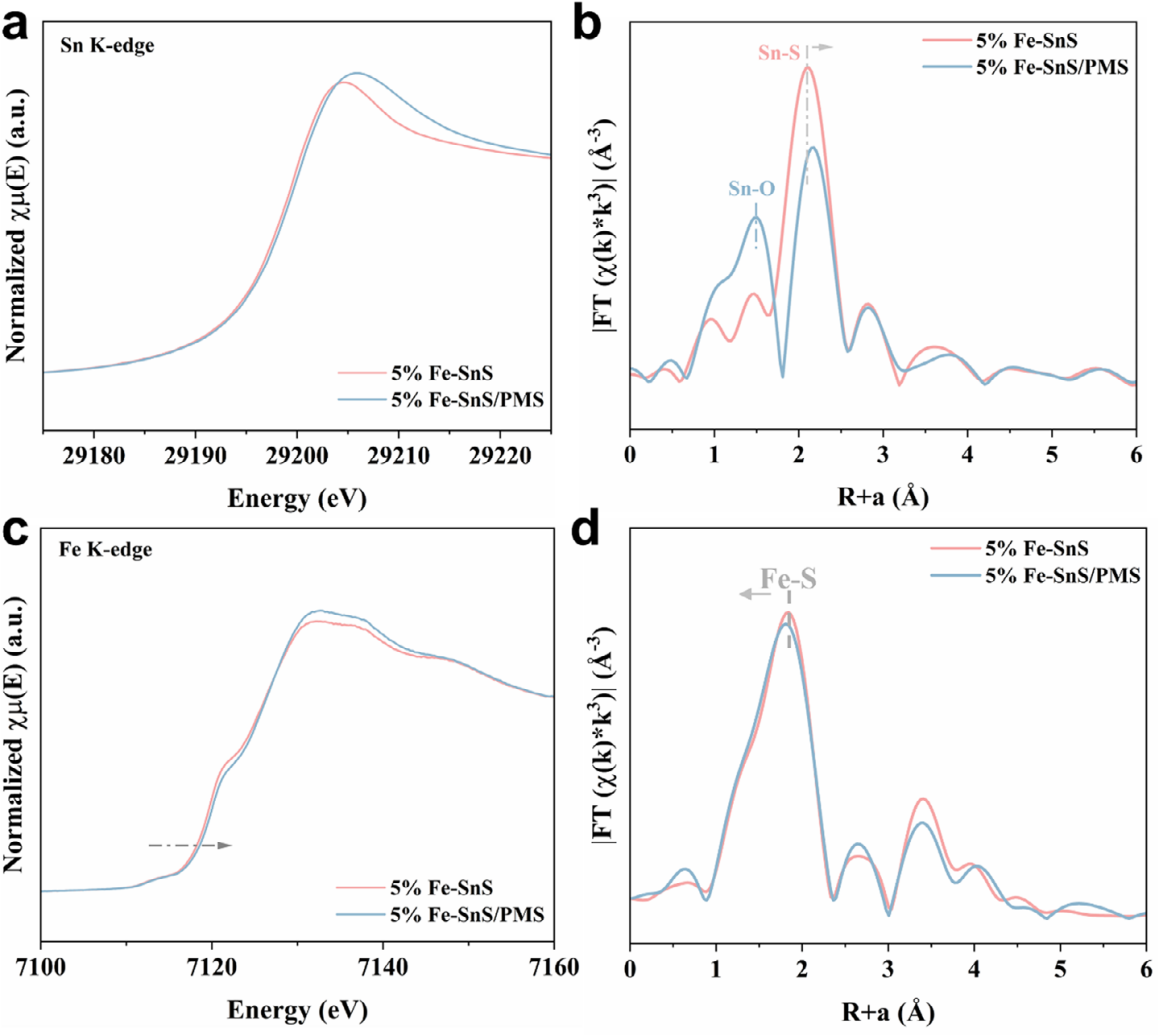
In Situ XAS analysis of Sn and Fe sites during PMS activation. (a) Normalized Sn K‐edge XANES spectra of 5% Fe‐SnS and 5% Fe‐SnS/PMS. (b) The corresponding Sn K‐edge EXAFS spectra of 5% Fe‐SnS before and after PMS addition at R space. (c) Normalized Fe K‐edge XANES spectra of 5% Fe‐SnS before and after PMS addition. (d) Fe K‐edge EXAFS analysis of 5% Fe‐SnS and 5% Fe‐SnS/PMS at R space.

Considering that pristine SnS showed some PMS activation activity and Fe incorporation further enhanced the catalytic performance, in situ XAS was performed to distinguish the roles of Sn and Fe sites during PMS activation by monitoring their local coordination and electronic states upon PMS addition. As shown in Figure 5a and Figure S32, the Sn K‐edge XANES spectra of 5% Fe‐SnS before and after PMS addition remain close to the SnS reference and are clearly distinct from the SnS_2_ reference, indicating that the average Sn electronic state remains predominantly Sn(II) upon PMS addition. The slight positive shift of the Sn K‐edge reflects PMS‐induced local electron depletion at Sn sites. Correspondingly, the FT‐EXAFS spectra show an attenuated and slightly shifted Sn‒S first‐shell peak together with the emergence of a new low‐R feature around 1.5 Å (Figure 5b). Supported by the WT‐EXAFS response (Figure S33), this low‐R feature can be assigned to Sn‒O scattering [45]. Quantitative Sn K‐edge EXAFS fitting further supports this local structural evolution. The first‐shell Sn‒S distances slightly increase from 2.51/2.62 Å to 2.54/2.65 Å after PMS addition, whereas the longer Sn‒S paths remain nearly unchanged at around 3.25 and 3.53 Å. Meanwhile, a Sn‒O‐related contribution with a fitted distance of ∼2.07 Å was obtained for the 5% Fe‐SnS/PMS (Figures S34 and S35, Table S6). These results indicate that PMS primarily perturbs the first‐shell coordination environment of Sn sites while the SnS framework is largely preserved. The additional Sn‒O scattering contribution further suggests a local interaction between Sn sites and O_PMS_ species, supporting the direct involvement of Sn sites in PMS interaction. Notably, upon PMS addition, pristine SnS also exhibits a comparable Sn–O contribution in the FT‐EXAFS spectrum (Figure S36), which is supported by the WT‐EXAFS analysis (Figure S37). EXAFS fitting confirms this coordination feature in the SnS/PMS system, yielding a fitted Sn–O distance of ∼2.07 Å (Figures S38 and S39, Table S7). These results show that PMS coordinates to Sn sites in both SnS and Fe‐SnS, indicating that Fe incorporation does not alter the predominant PMS coordination at Sn sites.

Meanwhile, the Fe sites exhibit a distinct response during PMS activation. Compared with Fe reference materials, the Fe K‐edge XANES spectra of 5% Fe‐SnS before and after PMS activation remain closer to that of FeO rather than Fe_2_O_3_, suggesting that Fe retains the Fe(II) electronic state during PMS activation (Figure S40). Upon PMS addition, the Fe K‐edge XANES spectrum shows a slight positive shift in the absorption edge and an increase in white‐line intensity, consistent with partial electron depletion at Fe sites and changes in their local electronic environment (Figure 5c) [46, 47, 48]. In contrast to the pronounced coordination response at Sn sites, the FT‐EXAFS spectra show that the dominant Fe‒S scattering feature is retained with minor R‐space variations (Figure 5d). Consistent with the first‐shell fitting of Fe K‐edge EXAFS [49, 50], nearly unchanged Fe‒S distances of 2.30 Å for 5% Fe‐SnS are detected before and after the addition of PMS (Figures S41 and S42, Table S8). Together with the WT‐EXAFS results, which reveal no pronounced Fe‒O feature (Figure S43), these results verify that Fe‒S coordination environment is largely preserved during PMS activation.

To further clarify the role of atomically dispersed Fe, charge‐density difference and Bader charge analysis were performed by DFT calculates. The charge density difference map reveals a highly localized redistribution of electron density around the Fe site and the adjacent Sn–S framework (Figure S44). Bader charge analysis shows that the Sn atoms adjacent to Fe are slightly electron‐enriched compared with the average Sn sites within Fe‐SnS, further supporting the Fe‐induced modulation of the local electronic environment of neighboring Sn centers (Table S9). Such Fe‐induced electronic redistribution can modify the interaction between neighboring Sn sites and PMS, thereby facilitating Sn‐centered PMS activation. For comparison, the initial PMS adsorption configurations on SnS and Fe‐SnS were constructed with the same terminal O atom of the O−O moiety directed toward a surface Sn site. After structural optimization, PMS forms more interfacial contacts with Fe‐SnS than with pristine SnS (Figure S45), suggesting that Fe incorporation facilitates the interfacial Sn−O_PMS_ interaction. The optimized O─O bond length remains nearly unchanged after Fe incorporation. Together with the charge‐density difference and Bader charge analyses, these results suggest that Fe incorporation facilitates Sn‐centered PMS activation through local electronic regulation and enhanced Sn–PMS interfacial interaction.

**SCHEME 1:**
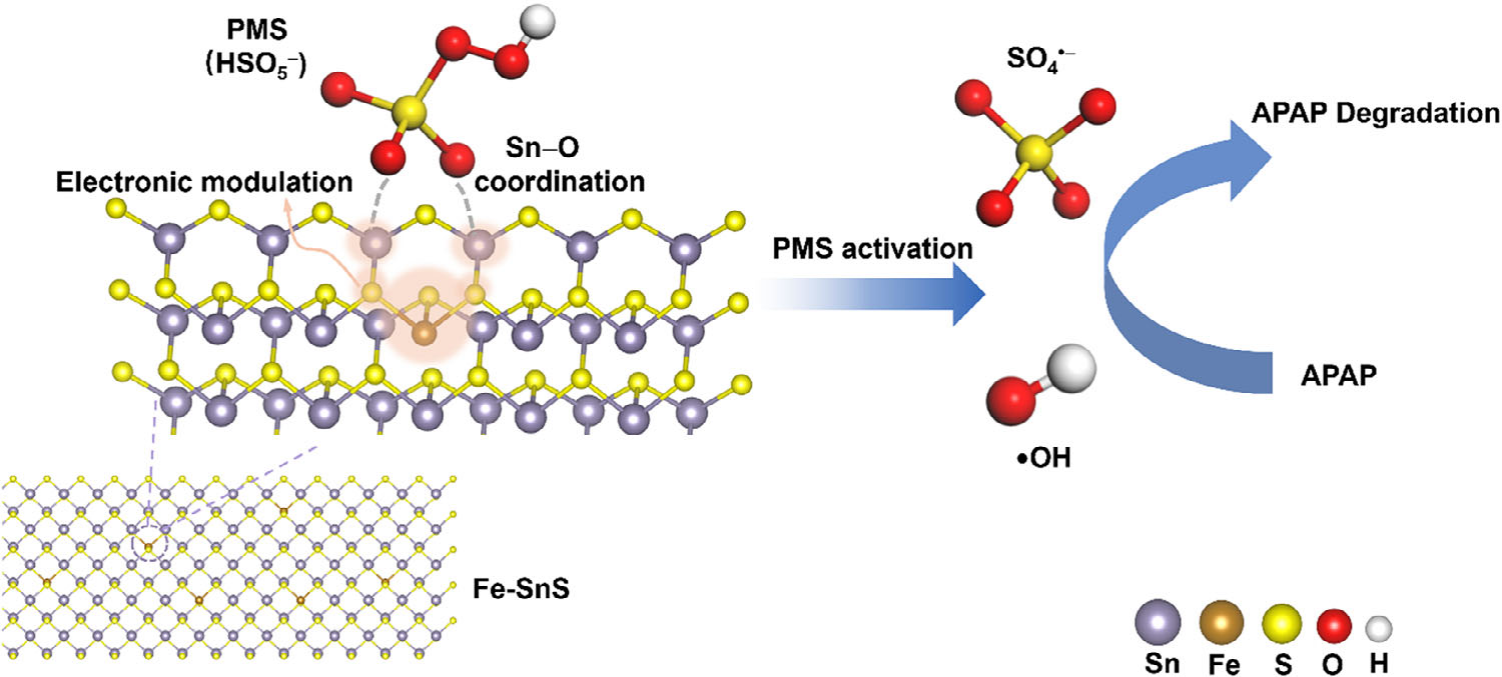
The proposed mechanism of PMS activation by the synergistic role of Sn and atomically dispersed Fe‒S sites in Fe‐SnS.

Overall, the in situ XAS/EXAFS results reveal distinct site‐dependent responses of Sn sites and atomically dispersed Fe during PMS activation over 5% Fe‐SnS. Sn sites exhibit the dominant coordination with PMS, as evidenced by the emergence of Sn–O scattering and the slight elongation of the first‐shell Sn–S distances. In contrast, the Fe–S coordination remains largely preserved, while Fe sites undergo a subtle electronic response during PMS activation. DFT calculations further show that Fe incorporation induces localized electronic redistribution around the neighboring Sn–S framework and enhances the Sn–PMS interfacial interaction. As illustrated in Scheme 1, this cooperative effect facilitates Sn‐centered PMS activation and the subsequent generation of SO_4_^•−^/•OH for APAP oxidation.

## 3 Conclusions

In summary, this study demonstrates that atomically dispersed Fe effectively regulates the local electronic environment and surface properties of SnS, leading to effective PMS activation for APAP oxidation. The introduced Fe is atomically dispersed as Fe‒S sites within SnS nanostructure. Mechanistic analyses by in situ XAS reveal that PMS preferentially interacts with Sn sites through the formation of Sn–O coordination and slight elongation of the first‐shell Sn–S bonds, whereas the Fe–S remains structurally preserved. DFT calculations further show that Fe introduction induces localized electronic redistribution around the Fe site and the neighboring Sn–S and enhances the Sn–PMS interfacial interaction. These findings support a Sn‐centered activation pathway in which atomically dispersed Fe promotes the reactivity of Sn sites through local electronic regulation and strengthened PMS interaction, leading to the generation of •OH and SO_4_^•−^ for APAP oxidation. This work provides a mechanistic basis for the atomic‐metal‐site engineering of metal chalcogenide catalysts.

## 4 Experimental Section

### 4.1 Chemical Reagents

Tin (II) chloride dihydrate (SnCl_2_·2H_2_O, 98%), thioacetamide (C_2_H_5_NS, 98%), iron(II) sulfate heptahydrate (FeSO_4_•7H_2_O) and urea (CH_4_N_2_O, 98%), potassium peroxymonosulfate (PMS, Oxone, >4%), Acetaminophen (APAP, 99%), methanol (CH_3_OH, 99%), tert‐Butanol (TBA, 99%), 2, 2, 6, 6‐tetramethyl‐4‐piperidinol, (TEMP, 98%), 5, 5‐dimethyl‐1‐pyrroline‐oxide (DMPO, 97%), methyl phenyl sulfoxide (PMSO, 97%), iron (II) sulfide (FeS, 99.9%), sodium sulfate (Na_2_SO_4_, 99%), sulfamethoxazole (SMX), bisphenol A (BPA, 99%), Rhodamine B (RhB, >95%), naproxen (NPX, 98%) were supplied by Sigma‐Aldrich. Tin disulphide (SnS_2_, >99.5%) and Tin(II) sulfide (SnS, 99%) were obtained from Aladdin platform. Ultrapure water was employed throughout the experiments. All chemicals in this work were used without further purification.

### 4.2 Measurements and Characterization

X‐ray powder diffraction (XRD, Rigaku MiniFlex 600) was implemented to confirm the crystal structure with 2‐theta degree from 20 to 80 at 40 kV and 15 mA. The morphology and structure of samples were analyzed by an Ultra‐High‐Resolution Schottky Scanning Electron Microscope (SEM, Hitachi SU7000) and the Spherical Aberration Corrected Transmission Electron Microscopy (TEM, Titan Themis FEI) at an acceleration voltage of 200 kV. Meanwhile, the high‐angle annular dark‐field scanning TEM (HAADF) and energy‐dispersive spectrometry mapping were captured at an acceleration voltage of 200 kV. Zeta potential was measured by Zetasizer Nano Series (Malvern). The ESR 5000 Spectrometer (Bruker, Germany) was used to detect the electron paramagnetic resonance (EPR) of the reactive oxygen species with 5,5‐dimethyl‐1‐pyrroline N‐oxide and 2,2,6,6‐tetramethyl‐4‐piperidinol as spin‐trapping agents. Fe loading (x, molar ratio) in Fe‐SnS was determined by inductively coupled plasma mass spectrometry (ICP‐MS). Atomic force microscopy (AFM) measurements were performed using an Asylum Research Cypher ES system, and the piezoelectric responses of SnS and 5% Fe‐SnS were further characterized by piezoresponse force microscopy (PFM) operated in contact mode. Total organic carbon (TOC) analysis was determined by TOC analyzer (Shimadzu, TOC‐L).

The X‐ray absorption spectra (XAS), including X‐ray absorption near‐edge structure (XANES) and extended X‐ray absorption fine structure (EXAFS) for the Fe K‐edge in Fe‐SnS were carried out on the beamline of X‐ray Absorption Spectroscopy in the Australian Synchrotron Center. FeO, FeS, FeSO_4_, Fe_2_O_3_, SnS standard and SnS_2_ were regarded as the standard references. All X‐ray absorption spectra of Fe K‐edge were measured in fluorescence mode under ambient conditions, while the X‐ray absorption spectra of Sn K‐edge were measured in a transmission mode under ambient conditions. The obtained spectra were energy calibrated and subsequently processed for background correction and edge step normalization by using ATHENA software. Raman spectra were investigated by Raman spectrometer (Horiba LabRAM HR Evolution) with a laser at 532 nm. X‐ray photoelectron spectroscopy (XPS) measurements were carried out to analyze the elemental composition and chemical status on an ESCALAB 250 Xi spectrometer (Thermo Fisher Scientific) fitted with Al Kα radiation. All the binding energies were referenced to the C 1s peak at 284.8 eV. High‐performance liquid chromatography (HPLC) was used to detect the residual concentration of pollutants after the catalytic degradation. The pH measurement and calibration were carried out on a METTLER TOLEDO Seven Direct SD 20 pH meter.

### 4.3 Electrochemical Tests

Open‐circuit potential (OCP) measurements of samples were conducted using a CHI760E electrochemical workstation, with a 3 mm diameter glassy carbon electrode served as the working electrode, Ag/AgCl functioned as the reference electrode, a platinum wire was employed as the counter electrode, and a 0.05 M Na_2_SO_4_ solution served as the electrolyte.

### 4.4 Synthesis of Fe‐SnS Nanobelts

Fe‐SnS was synthesized via a simple one‐pot hydrothermal method. In a typical procedure, SnCl_2_•2H_2_O and urea, thioacetamide were dissolved in deionized water, followed by the addition of different amounts of FeSO_4_•7H_2_O (corresponding to Fe/Sn molar ratios of 1%, 3%, 5%, and 10%). The mixture was stirred and ultrasonicated to ensure complete homogenization. The resulting solution was then transferred to a 100 mL Teflon‐lined stainless‐steel autoclave and heated at 170 °C for 10 h. After naturally cooling to room temperature, the precipitate was collected by filtration, washed three times with deionized water, and dried at 60 °C overnight. The final products were labeled as x% Fe‐SnS (x = 1, 3, 5, 10%), where x represents the nominal Fe/Sn molar ratio in the precursor solution, and used for further experiments. Pristine SnS was synthesized with a similar procedure without the addition of iron salt.

### 4.5 Experimental Procedures of Performance Activity

The performance of PMS activation by Fe‐SnS for organic pollutant degradation was evaluated by the degradation process of APAP. In this study, 20 µm APAP, pH 5.84, was used as the contaminant model. During the reaction process, the solution temperature was kept constant at 25°C. Firstly, added Fe‐SnS to APAP solution, sonicated for 1 min to make the catalysts dispersed homogeneously, and then left to stand for 30 min to establish the adsorption‐desorption equilibrium between the catalysts and APAP. Subsequently, the beaker was relocated to the constant temperature stirrer, where PMS was introduced to initiate the catalysis process. For comparison, control experiments included commercial SnS_2_ and SnS. At predetermined time intervals, 0.8 mL aliquots were drawn from the reaction suspension and filtered through 0.22 µm PTFE syringe filters to remove the residual catalyst. The residual ROS was quenched with methanol to terminate the catalytic reaction. The concentration of organic contaminants was analyzed by a high‐performance liquid chromatography (HPLC) system (Shimadzu) with the wavelength of the detector at 254 nm. The injection volume for HPLC analysis was 20 µL, and the mobile phases were methanol (30%) and aqueous acetic acid (70%) at a flow rate of 1.0 mL min^−1^. The removal efficiency was quantified in terms of the relative concentration (C_t_/C_0_) with the period, employing a pseudo‐first‐order kinetic model expressed as ln (C_t_/C_0_) = −k·t, where C_0_ and C_t_ denote the initial concentration and reaction concentration, and k represents the apparent rate constant. The degradation intermediates of APAP were analyzed by liquid chromatography‐mass spectrometry (LC‐MS, Thermo, Q Exactive Plus). Chromatographic separation was performed using a reversed‐phase C18 column with water (A) and methanol (B) as the mobile phases at a flow rate of 0.2 mL min^−1^. The gradient program was as follows: 0−10 min, A/B = 10:90 (v/v); 10−13 min, A/B = 90:10 (v/v); and 13.1−17 min, A/B = 10:90 (v/v). Electrospray ionization (ESI) was operated in both positive and negative ion modes for the identification of transformation products. All catalytic performance experiments were performed in duplicate (n = 2), and the results were reported as mean ± standard deviation (SD), with error bars representing the SD.

## Author Contributions

**Shuhui Liu**: methodology, investigation, writing – original draft, conceptualization. **Yantao Wang**: formal analysis, investigation. **Ya Liu**: formal analysis. **Jingkai Lin**: methodology, investigation. **Shaobin Wang**: supervision, funding acquisition, writing – review and editing. **Wenjie Tian**: conceptualization, supervision, funding acquisition, writing – review and editing.

## Funding

Discovery Project (FT250100204, DP230102406, and FL230100178) from the Australian Research Council.

## Conflicts of Interest

The authors declare no conflicts of interest.

## Supporting information




**Supporting File**: advs78025‐sup‐0001‐SuppMat.docx.

## Data Availability

The data that support the findings of this study are available from the corresponding author upon reasonable request.

## References

[1] A. Sengar and A. Vijayanandan , “Human Health and Ecological Risk Assessment of 98 Pharmaceuticals and Personal Care Products (PPCPs) Detected in Indian Surface and Wastewaters,” Science of the Total Environment 807 (2022): 150677, 10.1016/j.scitotenv.2021.150677. 34599960

[2] P. J. J. Alvarez , C. K. Chan , M. Elimelech , N. J. Halas , and D. Villagran , “Emerging Opportunities for Nanotechnology to Enhance Water Security,” Nature Nanotechnology 13 (2018): 634–641, 10.1038/s41565-018-0203-2. 30082804

[3] R. Du , L. Duan , Q. Zhang , et al., “Analysis on the Attenuation Characteristics of PPCPs in Surface Water and Their Influencing Factors Based on a Compilation of Literature Data,” Water Research 242 (2023): 120203, 10.1016/j.watres.2023.120203. 37336183

[4] H. Meng , J. Zhou , Y. Zhang , et al., “Single‐Atom Co‐N_3_ Sites Induce Peroxymonosulfate Activation for Acetaminophen Degradation via Nearly 100 % Internal Electron Transfer Process,” Applied Catalysis B: Environment and Energy 366 (2025): 125038, 10.1016/j.apcatb.2025.125038.

[5] S. Dewanjee , T. K. Dua , P. Paul , et al., “Probiotics: Evolving as a Potential Therapeutic Option Against Acetaminophen‐Induced Hepatotoxicity,” Biomedicines 10 (2022): 1498, 10.3390/biomedicines10071498. PMC9312935 35884803

[6] B. Ren , X. Shi , X. Jin , X. C. Wang , and P. Jin , “Comprehensive Evaluation of Pharmaceuticals and Personal Care Products (PPCPs) in Urban Sewers: Degradation, Intermediate Products and Environmental Risk,” Chemical Engineering Journal 404 (2021): 127024, 10.1016/j.cej.2020.127024.

[7] G. Liao , X. Qing , P. Xu , et al., “Synthesis of Single Atom Cobalt Dispersed on 2D Carbon Nanoplate and Degradation of Acetaminophen by Peroxymonosulfate Activation,” Chemical Engineering Journal 427 (2022): 132027, 10.1016/j.cej.2021.132027.

[8] W. Li , C. Nie , X. Wang , H. Ye , D. Li , and Z. Ao , “Alkaline Lignin‐Derived N‐Doped Biochars as Peroxymonosulfate Activators for Acetaminophen Degradation: Performance and Catalytic Bridging Mediated Electron‐Transfer Mechanism,” Separation and Purification Technology 323 (2023): 124418, 10.1016/j.seppur.2023.124418.

[9] B. C. Hodges , E. L. Cates , and J. H. Kim , “Challenges and Prospects of Advanced Oxidation Water Treatment Processes Using Catalytic Nanomaterials,” Nature Nanotechnology 13 (2018): 642–650, 10.1038/s41565-018-0216-x. 30082806

[10] H. Wu , X. Xu , L. Shi , et al., “Manganese Oxide Integrated Catalytic Ceramic Membrane for Degradation of Organic Pollutants Using Sulfate Radicals,” Water Research 167 (2019): 115110, 10.1016/j.watres.2019.115110. 31577967

[11] W. Tian , J. Lin , Z. Tian , et al., “Biomass Native Structure into Functional Carbon‐Based Catalysts for Fenton‐Like Reactions,” Advanced Functional Materials (2025): 2508759, 10.1002/adfm.202508759.

[12] B. Li , Y. Liu , K. Hu , et al., “Spin‐Regulated Fenton‐Like Catalysis for Nonradical Oxidation Over Metal Oxide@Carbon Composites,” Advanced Functional Materials 34 (2024): 2401397, 10.1002/adfm.202401397.

[13] S. Miao , H. Niu , Y. Wu , E. Fan , Y. Shen , and Q. Fang , “Regulating 4f‐2p‐3d Orbital Coupling in CeO(2) via Dual‐Transition Metal Doping for Efficient Peroxymonosulfate Activation,” Small 22 (2026): 14083, 10.1002/smll.202514083. 41589655

[14] X. Li , W. Xing , N. A. Al‐Dhabi , et al., “Encapsulated Nanoconfined Microenvironment and Fluorine Coordination Boost Selective Peroxymonosulfate Activation Toward Co(IV) = O Dominated High‐Efficiency Pollutant Degradation and Biotoxicity Abatement,” Advanced Functional Materials 36 (2026): 75641, 10.1002/adfm.75641.

[15] T. Yu , N. A. Al‐Dhabi , W. Xing , et al., “Highly Efficient Antibiotic Decontamination and Biotoxicity Elimination With Activity‐Stability Considerations via Encapsulated Catalyst Induced Peroxymonosulfate Activation,” Journal of Environmental Management 396 (2025): 128078, 10.1016/j.jenvman.2025.128078. 41275777

[16] Y. Yan , Z. Wei , X. Duan , et al., “Merits and Limitations of Radical vs. Nonradical Pathways in Persulfate‐Based Advanced Oxidation Processes,” Environmental Science & Technology 57 (2023): 12153–12179, 10.1021/acs.est.3c05153. 37535865

[17] Q. Tian , Y. Jiang , X. Duan , Q. Li , Y. Gao , and X. Xu , “Low‐Peroxide‐Consumption Fenton‐Like Systems: The Future of Advanced Oxidation Processes,” Water Research 268 (2025): 122621, 10.1016/j.watres.2024.122621. 39426044

[18] W. Tian , N. Li , D. Chen , et al., “Vibration‐Driven Reduction of CO_2_ to Acetate With 100% Selectivity by SnS Nanobelt Piezocatalysts,” Angewandte Chemie International Edition 62 (2023): 202306964, 10.1002/anie.202306964. 37287329

[19] H. Khan , N. Mahmood , A. Zavabeti , et al., “Liquid Metal‐Based Synthesis of High Performance Monolayer SnS Piezoelectric Nanogenerators,” Nature Communications 11 (2020): 3449, 10.1038/s41467-020-17296-0. PMC7351749 32651367

[20] Q. Xie , H. Zhou , Z. Lv , H. Liu , and H. Guo , “Sn4+ Self‐Doped Hollow Cubic SnS as an Efficient Visible‐Light Photocatalyst for Cr(vi) Reduction and Detoxification of Cyanide,” Journal of Materials Chemistry A 5 (2017): 6299–6309, 10.1039/c6ta11052e.

[21] Y. Shan , Y. Li , and H. Pang , “Applications of Tin Sulfide‐Based Materials in Lithium‐Ion Batteries and Sodium‐Ion Batteries,” Advanced Functional Materials 30 (2020): 2001298, 10.1002/adfm.202001298.

[22] A. de Kergommeaux , M. Lopez‐Haro , S. Pouget , et al., “Synthesis, Internal Structure, and Formation Mechanism of Monodisperse Tin Sulfide Nanoplatelets,” Journal of the American Society 137 (2015): 9943–9952, 10.1021/jacs.5b05576. 26200758

[23] Z. Tian , C. Guo , M. Zhao , R. Li , and J. Xue , “Two‐Dimensional SnS: A Phosphorene Analogue With Strong In‐Plane Electronic Anisotropy,” ACS Nano 11 (2017): 2219–2226, 10.1021/acsnano.6b08704. 28106983

[24] M. Xu , J. Wang , L. Zhang , et al., “Synergistic Piezo‐Catalytic Degradation of Organic Pollutants and Cr (VI) by Co‐Metallene/SnS Nanorods: Mechanistic Insights,” Applied Surface Science 690 (2025): 162536, 10.1016/j.apsusc.2025.162536.

[25] Q. Xia , B. Huang , X. Yuan , et al., “Modified Stannous Sulfide Nanoparticles With Metal‐Organic Framework: Toward Efficient and Enhanced Photocatalytic Reduction of Chromium (VI) Under Visible Light,” Journal of Colloid and Interface Science 530 (2018): 481–492, 10.1016/j.jcis.2018.05.015. 29990784

[26] S. Lan , B. Jing , C. Yu , et al., “Protrudent Iron Single‐Atom Accelerated Interfacial Piezoelectric Polarization for Self‐Powered Water Motion Triggered Fenton‐Like Reaction,” Small 18 (2022): 2105279, 10.1002/smll.202105279. 34837320

[27] Y. Sun , R. Li , C. Song , et al., “Origin of the Improved Reactivity of MoS_2_ Single Crystal by Confining Lattice Fe Atom in Peroxymonosulfate‐Based Fenton‐Like Reaction,” Applied Catalysis B: Environmental 298 (2021): 120537, 10.1016/j.apcatb.2021.120537.

[28] Z. Yang , X. Yang , G. An , and D. Wang , “Regulating Spin state of Fe Active Sites by the P‐Doping Strategy for Enhancing Peroxymonosulfate Activation,” Applied Catalysis B: Environmental 330 (2023): 122618, 10.1016/j.apcatb.2023.122618.

[29] H. Li , X. Qin , K. Wang , T. Ma , and Y. Shang , “Insight Into Metal‐Based Catalysts for Heterogeneous Peroxymonosulfate Activation: A Critical Review,” Separation and Purification Technology 333 (2024): 125900, 10.1016/j.seppur.2023.125900.

[30] K. Zhu , W. Qin , Y. Gan , et al., “Acceleration of Fe^3+^/Fe^2+^ Cycle in Garland‐Like MIL‐101(Fe)/MoS_2_ Nanosheets to Promote Peroxymonosulfate Activation for Sulfamethoxazole Degradation,” Chemical Engineering Journal 470 (2023): 144190, 10.1016/j.cej.2023.144190.

[31] H. Li , P. Zhang , Y. Guo , et al., “Iron‐Doped Cuprous Oxides Toward Accelerated Nonradical Oxidation: Doping Induced Controlled Facet Transformation and Optimized Electronic Structure,” Chemical Engineering Journal 407 (2021): 127172, 10.1016/j.cej.2020.127172.

[32] R. Jiang , G. Lu , M. Wang , et al., “Lattice Distortion SnS_2_ Piezoelectric Self‐Fenton System for Efficient Degradation and Detoxification of Pollutants,” npj Clean Water 6 (2023): 79, 10.1038/s41545-023-00293-3.

[33] J. Wang , B. Li , Y. Li , et al., “Facile Synthesis of Atomic Fe‐N‐C Materials and Dual Roles Investigation of Fe‐N(4) Sites in Fenton‐Like Reactions,” Advanced Science 8 (2021): 2101824, 10.1002/advs.202101824. PMC8596112 34643069

[34] Q. Jiang , Y. Ma , P. Zhao , X. Li , Y. Shao , and X. Xu , “Electronic Structure Regulation of Fe Sites by Coordinating Moieties in the Fenton‐Like Process Enables Tunable Water Decontamination,” Environmental Science & Technology 59 (2025): 14182–14192, 10.1021/acs.est.5c06061. 40605236

[35] S. Liu , H. Zhang , Y. Wang , H. Sun , S. Wang , and W. Tian , “Morphology‐Enhanced Piezoelectric Performance of SnS Nanobelts for Acetaminophen Degradation,” Journal of Materials Chemistry A 12 (2024): 15744–15752, 10.1039/d4ta02531h.

[36] X. Jiang , B. Zhou , W. Yang , et al., “Precise Coordination of High‐Loading Fe Single Atoms With Sulfur Boosts Selective Generation of Nonradicals,” Proceedings of the National Academy of Sciences of the United States of America 121 (2024): 2309102121, 10.1073/pnas.2309102121. PMC10823248 38232287

[37] P. Zhang , Y. Yu , K. Li , et al., “Trifunctional P‐Doping of FeS_1‐x_ for Greatly Enhanced Electrochemical Kinetics and Highly Resilient Li‐S Batteries,” Advanced Energy Materials 16 (2025): 2501940, 10.1002/aenm.202501940.

[38] F. Meng , C. Guo , T. Cui , et al., “Rapid Synthesis of Single‐Layer Iron‐Doped 2H Tungsten Sulfide via Magnetic Induction Heating for Piezocatalytic Reduction of Oxygen to Hydrogen Peroxide,” Applied Catalysis B: Environment and Energy 371 (2025): 125224, 10.1016/j.apcatb.2025.125224.

[39] J. Lin , K. Hu , Y. Wang , et al., “Tandem Microplastic Degradation and Hydrogen Production by Hierarchical Carbon Nitride‐Supported Single‐Atom Iron Catalysts,” Nature Communications 15 (2024): 8769, 10.1038/s41467-024-53055-1. PMC11464750 39384850

[40] L. Cui , X. Zhao , H. Xie , and Z. Zhang , “Overcoming the Activity–Stability Trade‐Off in Heterogeneous Electro‐Fenton Catalysis: Encapsulating Carbon Cloth‐Supported Iron Oxychloride Within Graphitic Layers,” ACS Catalysis 12 (2022): 13334–13348, 10.1021/acscatal.2c03571.

[41] Z. S. Zhu , Y. Wang , X. Duan , et al., “Atomic‐Level Engineered Cobalt Catalysts for Fenton‐Like Reactions: Synergy of Single Atom Metal Sites and Nonmetal‐Bonded Functionalities,” Advanced Materials 36 (2024): 2401454, 10.1002/adma.202401454. 38685794

[42] C. Cheng , W. Ren , F. Miao , X. Chen , X. Chen , and H. Zhang , “Generation of Fe(IV) = O and Its Contribution to Fenton‐Like Reactions on a Single‐Atom Iron‐N‐C Catalyst,” Angewandte Chemie International Edition 62 (2023): 202218510, 10.1002/anie.202218510. 36625681

[43] J. Yao , N. Wu , X. Tang , Z. Wang , R. Qu , and Z. Huo , “Methyl Phenyl Sulfoxide (PMSO) as a Quenching Agent for High‐Valent Metal‐Oxo Species in Peroxymonosulfate Based Processes Should be Reconsidered,” Chemical Engineering Journal Advances 12 (2022): 100378, 10.1016/j.ceja.2022.100378.

[44] Y. Gao , Y. Zhou , S.‐Y. Pang , Z. Wang , Y.‐M. Shen , and J. Jiang , “Quantitative Evaluation of Relative Contribution of High‐Valent Iron Species and Sulfate Radical in Fe(VI) Enhanced Oxidation Processes via Sulfur Reducing Agents Activation,” Chemical Engineering Journal 387 (2020): 124077, 10.1016/j.cej.2020.124077.

[45] Y. Ji , H. Zhou , S. Liu , et al., “Isolating Single Sn Atoms in CuO Mesocrystal to Form Ordered Atomic Interfaces: An Effective Strategy for Designing Highly Efficient Mesocrystal Catalysts,” Small 18 (2022): 2203658, 10.1002/smll.202203658. 36161498

[46] T. Chen , G. Zhang , H. Sun , et al., “Robust Fe‐N(4)‐C(6)O(2) Single Atom Sites for Efficient PMS Activation and Enhanced Fe(IV) = O Reactivity,” Nature Communications 16 (2025): 2402, 10.1038/s41467-025-57643-7. PMC11894199 40064929

[47] D. Zhang , Y. Li , P. Wang , J. Qu , Y. Li , and S. Zhan , “Dynamic Active‐Site Induced by Host‐Guest Interactions Boost the Fenton‐Like Reaction for Organic Wastewater Treatment,” Nature Communications 14 (2023): 3538, 10.1038/s41467-023-39228-4. PMC10272134 37322015

[48] S. Ren , Y. Wang , L. Shi , et al., “Transforming Plastics to Single Atom Catalysts for Peroxymonosulfate Activation: Axial Chloride Coordination Intensified Electron Transfer Pathway,” Advanced Materials 37 (2025): 2415339, 10.1002/adma.202415339. 39757509

[49] D. Mitra , S. J. George , Y. Guo , et al., “Characterization of [4Fe‐4S] Cluster Vibrations and Structure in Nitrogenase Fe Protein at Three Oxidation Levels via Combined NRVS, EXAFS, and DFT Analyses,” Journal of the American Society 135 (2013): 2530–2543, 10.1021/ja307027n. PMC3644515 23282058

[50] A. Matamoros‐Veloza , O. Cespedes , B. R. G. Johnson , et al., “A Highly Reactive Precursor in the Iron Sulfide System,” Nature Communications 9 (2018): 3125, 10.1038/s41467-018-05493-x. PMC6081449 30087338

