# Supplementary material for: Atomically Dispersed Fe‐Modified SnS for Sn‐Centered Peroxymonosulfate Activation Toward Acetaminophen Degradation
Source: Adv Sci (Weinh). 2026 Sep 27:e78025. Online ahead of print. doi: 10.1002/advs.78025 (PMC13616257; doi:10.1002/advs.78025)
Supplement: Supplementary file 1 [file ADVS-9999-e78025-s001.docx]

Supporting Information

Atomically Dispersed Fe-Modified SnS for Sn-Centered Peroxymonosulfate Activation toward Acetaminophen Degradation

Shuhui Liu, Yantao Wang, Ya Liu, Jingkai Lin, Shaobin Wang*, Wenjie Tian*

School of Chemical Engineering, Adelaide University, Adelaide, SA, 5005, Australia

*Corresponding authors: shaobin.wang@adelaide.edu.au; wenjie.tian@adelaide.edu.au

**Text S1.** Semi-quantitative estimation of the relative contributions of •OH and SO_4_^•−^.

The k_obs_ values obtained from the quenching experiments were used to provide a semi-quantitative estimate of the relative contributions of •OH and SO_4_^•−^ to APAP degradation.[1] The apparent rate constants were determined from the initial pseudo-first-order kinetic fits. Here, k_0_ denotes the rate constant of the reaction in the absence of any scavenger, whereas k_1_ and k_2_ denote the rate constants obtained in the presence of TBA and methanol, respectively. Given that TBA preferentially scavenges •OH, whereas methanol effectively scavenges both •OH and SO_4_^•−^, their relative contributions were calculated using Equations (1) and (2):

λ[•OH] = (k_0_-k_1_)/K_0_	(1)

λ[SO_4_^•−^] = (k_1_-k_2_)/K_0_	(2)

Text S2. Theoretical calculations.

Density functional theory (DFT) calculations were performed by using the Vienna ab initio simulation package (VASP).[2-3] The electron-ion potential was described by the projected augmented wave method (PAW),[4] and a plane-wave energy cut-off of 450 eV was set in this study. The Perdew-Burk-Ernzerhof (PBE) within the generalized gradient approximation (GGA) was used to describe the exchange-correlation functional.[5] The convergence criterion of the force was -0.03 eV/Å and the convergence thresholds for the total energy was 10^-5^ eV. The Brillouin-zone integration was approximated 4×4×1 by grid using the Monkhorst-Pack k-point mesh. A vacuum space of 15 Å in the Z-direction was chosen to avoid artificial interaction. The effect of van der Waals (vdW) interactions was included for weak interaction cases using the semiempirical correction scheme of Becke-Jonson damping, DFT-D3.[6]

**Figure S1.** The XRD patterns of SnS and Fe-SnS samples.

**Figure S2.** (a) The XRD pattern of Fe_1-x_S synthesized without Sn precursor. (b) XRD comparison between Fe_1-x_S and Fe-SnS with high Fe content (10% Fe-SnS).

**Figure S3.** SEM images of (a) SnS, (b) 1% Fe-SnS, (c) 3% Fe-SnS.

**Figure S4.** The HAADF images of (a) 1% Fe-SnS and (b) 3% Fe-SnS.

**Figure S5.** AFM topographic and PFM images of SnS. (a) 3D AFM topographic image, (b) AFM height image, (c) PFM amplitude image, and (d) PFM phase image of SnS.

**Figure S6.** AFM topographic and PFM images of 5% Fe-SnS. (a) 3D AFM topographic image, (b) AFM height image, (c) PFM amplitude image, and (d) PFM phase image of 5% Fe-SnS.

**Figure S7.** Phase hysteresis loop and amplitude-voltage butterfly curves of (a) SnS and (b) 5% Fe-SnS. Pink symbols correspond to amplitude response, while blue symbols correspond to phase response.

**Figure S8**. The HAADF-STEM images of 5% Fe-SnS (a) morphology, (b) atomic resolution collected from a particle attached to the nanobelt.

**Figure S9.** WT-EXAFS for Fe K-edge for (a) FeO, (b) FeS, and (c) FeSO_4_ references.

**Figure S10**. WT-EXAFS for Sn K-edge for SnS.

**Figure S11**. WT-EXAFS for Sn K-edge for (a) SnS standard, (b) SnS_2_ standard reference.

**Figure S12.** XPS spectra of 5% Fe-SnS (a) Sn 3d, (b) S 2p regions.

The Sn 3d X-ray photoelectron spectroscopy (XPS) spectrum of 5% Fe-SnS (**Figure S12a**) shows two main peaks at 494.8 and 486.4 eV, corresponding to Sn 3d_3/2_ and Sn 3d_5/2_ of Sn^2+^.[7] Additional weaker signals at 496.1 and 487.6 eV are attributed to Sn^4+^ species, likely originating from surface oxidation upon air exposure.[8] The high-resolution S 2p spectrum (**Figure S12b**) exhibits two distinct peaks at 161.8 and 163.0 eV, corresponding to S 2p_3/2_ and S 2p_1/2_ of S species, respectively.[9] The Fe 2p XPS region overlaps with the Sn 3p signal, which introduces uncertainty in peak deconvolution. Therefore, Fe valence and coordination were primarily determined by XAS.

**Figure S13.** (a) Degradation of APAP in 5% Fe-SnS/PMS system with different catalyst dosages. (b) Pseudo-first-order kinetic fitting of the corresponding reactions. Experimental conditions: [PMS] = 0.5 mM, [APAP] = 20 μM, T = 25 ℃.

**Figure S14.** APAP adsorption rates of Fe-SnS with different Fe loading ratios. Experimental conditions: [APAP] = 20 μM, [catalysts] = 0.4 g L^−1^, T = 25 ℃.

**Figure S15.** The corresponding pseudo-first-order kinetic constant (k) in different reaction systems. Experimental conditions: [PMS] = 0.5 mM, [APAP] = 20 μM, [catalyst] = 0.4 g L^−1^, T = 25 ℃.

**Figure S16.** (a) Degradation efficiency of APAP with different reaction systems. (b) The corresponding reaction rate constants in different reaction systems. Degradation kinetics data were fitted using a first-order equation. (c) Comparison of degradation efficiencies and reaction rate constants of commercial SnS, commercial SnS_2_, SnS and 5% Fe-SnS. Experimental conditions: [Catalyst] = 0.4 g L^−1^, [PMS] = 0.5 mM, [APAP] = 20 μM, T = 25 °C.

**Figure S17.** APAP degradation comparison in PMS, homogeneous Fe^2+^/PMS, and 5% Fe-SnS/PMS systems. Experimental conditions: [PMS] = 0.5 mM, [APAP] = 20 μM, [5% Fe-SnS] = 0.4 g L^−1^, [Fe^2+^] = 5.52 μM, T = 25 ℃.

**Figure S18.** Catalytic performance comparison of Fe_1-x_S, SnS, and 5% Fe-SnS for APAP degradation. Experimental conditions: [SnS] =[5% Fe-SnS] =  0.4 g L^−1^, [Fe_1-x_S] = 0.4 mg, [PMS] = 0.5 mM, [APAP] = 20 μM, T = 25 °C.

**Figure S19.** Degradation of different organic pollutants in the 5% Fe-SnS/PMS system. Experimental conditions: [pollutants] = 20 μM, [PMS] = 0.5 mM, [catalyst] = 0.4 g L^−1^, T = 25 °C.

Beyond APAP, the applicability of the 5% Fe-SnS/PMS system was also assessed using several representative emerging contaminants. As shown in Figure S19, SMX and NPX were nearly completely degraded within 10 min, while the degradation efficiencies of BPA and RhB reached approximately 95% and 74%, respectively. These results demonstrate the broad applicability of the Fe-SnS/PMS system toward various emerging contaminants.

**Figure S20.** APAP degradation in 5% Fe-SnS/PMS system with different pH values. Experimental conditions: [Catalysts] = 0.4 g L^−1^, [PMS] = 0.5 mM, [APAP] = 20 μM, T = 25 ℃.

**Figure S21.** Influence of inorganic ions and HA on the degradation of APAP in the 5% Fe-SnS/PMS system. Experimental conditions: [PMS] = 0.5 mM, [catalyst] = 0.4 g L^−1^, [APAP] = 20 μM, [inorganic ions] = 1 mmol L^−1^, [HA]= 1 ppm, T = 25 °C.

Considering the potential influence of water constituents on AOPs, the effects of Cl^−^, HCO_3_^−^, HPO_4_^2−^, and humic acid (HA) were also examined (Figure S21). Cl^−^ and HA did not inhibit APAP degradation, whereas HCO_3_^−^ and HPO_4_^2−^ markedly suppressed APAP degradation. Such inhibition is consistent with previous PMS-based systems, where HCO_3_^−^ can scavenge •OH and SO_4_^•−^ to form less reactive carbonate radicals, while HPO_4_^2−^ can interfere with radical reactions and PMS activation at the catalyst surface.[10-11] Despite these matrix effects, APAP was almost completely removed in tap water within 10 min.

**Figure S22.** Total of organic carbon in the solution before and after the reaction. Experimental conditions: [Catalysts] = 0.4 g L^−1^, [PMS] = 0.5 mM, [APAP] = 20 μM, T = 25 ℃.

Despite the nearly complete removal of APAP, the TOC concentration remained essentially unchanged after 10 min (Figure S22), revealing limited mineralization and indicating that APAP was primarily converted into organic transformation products. To further elucidate the degradation process, the major transformation products were subsequently identified by LC–MS.

**Figure S23.** Proposed degradation pathways of APAP in the 5% Fe-SnS/PMS system based on the transformation products tentatively identified by LC–MS.

**Figure S24.** ECOSAR-predicted acute (blue plots) and chronic (yellow plots) aquatic ecotoxicities of APAP and its major transformation products toward fish, daphnids, and green algae. (Neutral Organics predictions were used for comparison, with Neutral Organics-acid predictions used for acid-containing products. The name of these products was shown in Table S5).

**Figure S25.** Recycling test and post-reaction characterization of 5% Fe-SnS. (a) Recycling performance for APAP degradation. (b) XRD patterns of 5% Fe-SnS before and after reaction. High-resolution XPS spectra of 5% Fe-SnS after reaction (c) Sn 3d and (d) S 2p, respectively.

**Figure S26.** PMSO consumption and PMSO_2_ generation in the 5% Fe-SnS/PMS system. Experimental conditions: [PMS] = 0.5 mM, [catalyst] = 0.4 g L^−1^, [PMSO] = 1 mmol L^−1^, T = 25 °C.

**Figure S27.** Corresponding reaction rate constants for APAP degradation by using different scavengers. Experimental conditions: [PMS] = 0.5 mM, [catalyst] = 0.4 g L^−1^, [APAP] = 20 μM, [TBA] = [Methanol] = 200 mM, [TEMP] = [PMSO] = 5 mM, T = 25 °C.

**Figure S28.** EPR spectra of ^1^O_2_ in 5% Fe-SnS/PMS system.

**Figure S29.** Open-circuit potential curves with the addition of 0.5 mM PMS on (a) Blank electrode and (b) 5% Fe-SnS as working electrode. Experimental conditions: [Na_2_SO_4_] = 0.05 M, [PMS] = 0.5 mM. [APAP]= 20 μM.

Upon PMS addition, only a slight increase in the OCP was observed on the 5% Fe-SnS electrode, and no apparent potential drop was observed after the introduction of APAP.

**Figure S30.** In-situ Raman spectra of PMS activation with 5% Fe-SnS.

As shown in Figure S30, the characteristic peaks at 881 and 1058 cm^−1^ were observed, corresponding to the bending and stretching vibrations of the HSO_5_^−^ species, while the peak at 979 cm^−1^ is attributed to the stretching vibration of the S=O in SO_4_^2−^.[12-13] After the introduction of 5% Fe-SnS, no new Raman signals or significant peak shifts were detected, indicating that no active peroxide (PMS*) or electron transfer pathway was involved in this system.

**Figure S31.** Zeta potential analysis of SnS and Fe-SnS with varying Fe contents. (a) Zeta potential of SnS and 5% Fe-SnS. (b) Trend of the zeta potential of the samples with increasing Fe content ratio.

Zeta-potential measurements were conducted with the samples dispersed in ultrapure water without pH adjustment or the addition of PMS or electrolytes. All samples were measured under identical conditions to compare their relative surface-charge characteristics.

**Figure S32.** In situ Sn K-edge XANES spectra of 5% Fe-SnS before and after PMS addition, compared with SnS and SnS_2_ standard references.

**Figure S33.** Sn K-edge WT-EXAFS plots of (a) 5% Fe-SnS, (b) 5% Fe-SnS/PMS.

**Figure S34.** Sn K-edge EXAFS (points) and the curve fit (line) for 5% Fe-SnS in (a) k space, (b) R-space (FT magnitude and imaginary component).

**Figure S35.** Sn K-edge EXAFS (points) and the curve fit (line) for 5% Fe-SnS/PMS in (a) k space, (b) R-space (FT magnitude and imaginary component).

**Figure S36.** *In situ* XAS analysis of Sn site during PMS activation. (a) Normalized Sn K-edge XANES spectra of SnS and SnS/PMS. (b) The corresponding Sn K-edge EXAFS spectra of SnS before and after PMS addition in R space.

**Figure S37.** Sn K-edge WT-EXAFS plots of (a) SnS, (b) SnS/PMS.

**Figure S38.** Sn K-edge EXAFS (points) and the curve fit (line) for SnS in (a) k space, (b) R-space (FT magnitude and imaginary component).

**Figure S39.** Sn K-edge EXAFS (points) and the curve fit (line) for SnS/PMS in (a) k space, (b) R-space (FT magnitude and imaginary component).

**Figure S40.** *In situ* Fe K-edge XANES spectra of 5% Fe-SnS before and after PMS addition.

**Figure S41.** Fe K-edge EXAFS (points) and the curve fit (line) for 5% Fe-SnS in (a) k space, (b) R-space (FT magnitude and imaginary component).

**Figure S42.** Fe K-edge EXAFS (points) and the curve fit (line) for 5% Fe-SnS/PMS in (a) k space, (b) R-space (FT magnitude and imaginary component).

**Figure S43.** Fe K-edge WT-EXAFS spectra of (a) 5% Fe-SnS, (b) 5% Fe-SnS after PMS addition.

**Figure S44.** Charge density difference of Fe-SnS, showing the local charge redistribution induced by Fe incorporation. Yellow and cyan isosurfaces denote electron accumulation and depletion, respectively, at an isosurface value of 0.0037 e Å^−3^.

**Figure S45.** Optimized adsorption configurations of PMS on (a) SnS and (b) Fe-SnS.

**Table S1.** Inductively coupled plasma mass spectrometry (ICP-MS) results for Fe content in Fe-SnS (the molar ratio of SnS)

| Samples | 1% Fe-SnS | 3% Fe-SnS | | 5% Fe-SnS | 10% Fe-SnS |
| --- | --- | --- | --- | --- | --- |
| The molar ratio of n(Fe)/n(Sn) | 1.64% | 3.89% | 4.63% | | 8.87% |

**Table S2.** ICP-determined concentrations of Fe and Sn leaching in the post-reaction solution.

| Sample name | Element | Concentration (μg L^−1^) |
| --- | --- | --- |
| 5% Fe-SnS | Fe | 308.51 |
| 5% Fe-SnS | Sn | 34.33 |

**Table S3.** Summary of acetaminophen degradation in PMS activation systems.

| Catalysts | Catalysts dosage (g/L) | PMS dosage (mM) | Kobs (min^-1^) | pH | Performance | Main active species | Ref. |
| --- | --- | --- | --- | --- | --- | --- | --- |
| Fe^2+^/MoS_2_ | 0.1 | 0.5 | 0.1099 | 3 | 94.5% degradation of APAP in 180 min | ^1^O_2_, •OH, •SO_4_^−^, | ^[14]^ |
| Fe(III)/Cu(0) | 0.2 | 1.5 | 0.1767 | 3 | 88.8% APAP degradation by in 12 min | Fe(IV), •OH | ^[15]^ |
| Carbon material (CMN) | 0.1 | 0.662 | 0.3677 | 6.5 | 95% removal of APAP within 30 min | ^1^O_2_, •OH, •SO_4_^−^, O_2_^•−^ | ^[16]^ |
| Chlorine | 0.1 mM | 1 | 0.2899 | 7 | 86.0% degradation of APAP | ^1^O_2_, •OH, •SO_4_^−^, Cl• | ^[17]^ |
| S-MgO | 1 | 1.63 | 0.314 | 7 | 95.6% removal of APAP in 30 min | •OH, •SO_4_^−^ | ^[18]^ |
| Na_2_B_4_O_7_/heat | 10 mM | 1.5 | 0.298 | 9 | 100% degradation of APAP in 15min | ^1^O_2_, •OH, •SO_4_^−^ | ^[19]^ |
| MgO_2_ | 0.169 | 3 | 0.446 | - | 100% degradation of APAP within 60 min | ^1^O_2_, O_2_^•−^ | ^[20]^ |
| Fe_3_O_4_ | 0.8 | 0.2 | 0.039 | 4.3 | 100% degradation of APAP in 2h | •OH, •SO_4_^−^, | ^[21]^ |
| N/S Co-doped mesoporous carbon | 0.1 | 0.5 | 0.246 | 5-9 | 100% degradation of APAP in 30 min | ^1^O_2_, •OH, •SO_4_^−^, | ^[22]^ |
| CoNBC600 | 0.1 | 0.5 | 0.46 | 5.6 | 100% degradation of APAP in 15 min | ^1^O_2_ | ^[23]^ |
| SA-Co CNP | 0.2 | 3.29 | 0.51 | 7 | 92% degradation of APAP within 10 min | ^1^O_2_ | ^[24]^ |
| Mn@NBC-800 | 0.02 | 1 | 0.438 | 3-5 | 100% degradation of APAP in 10 min | ^1^O_2_ | ^[25]^ |
| Co-SnP | 0.2 | 0.5 | 0.282 | 7 | nearly complete degradation of APAP within 15 min | •OH, •SO_4_^−^ | ^[26]^ |
| Coconut shell biochar | 0.5 | 1.1 | 0.107 | 7 | 99% removal of APAP in 30 min | ^1^O_2_, O_2_^•−^ | ^[27]^ |
| 5% Fe-SnS | 0.4 | 0.5 | 1.23 | 5.8 | 99% removal of APAP in 10min | •OH, •SO_4_^−^ | This work |

**Table S4.** HPLC-MS information for APAP and its major transformation products in the 5% Fe-SnS/PMS system.

**Table S5.** Predicted acute and chronic ecotoxicities of APAP and its major transformation products toward fish, daphnids, and green algae using ECOSAR.

| **Name** | **Formula** | **Fish LC_50_ (mg/L)** | **Daphnid LC_50_ (mg/L)** | **Algae EC_50_ (mg/L)** | **Fish ChV (mg/L)** | **Daphnid ChV mg/L** | **Algae ChV (mg/L)** |
| --- | --- | --- | --- | --- | --- | --- | --- |
| P-1 | C_8_H_9_NO_2_ | 3000.054 | 1477.661 | 611.425 | 247.963 | 97.009 | 116.66 |
| P-2 | C_8_H_9_NO_3_ | 6439.349 | 3079.128 | 1127.259 | 513.962 | 186.155 | 201.355 |
| P-3 | C_6_H_7_NO | 3379.747 | 1631.926 | 621.997 | 272.875 | 101.373 | 113.54 |
| P-4 | C_6_H_7_NO_2_ | 10460.867 | 4832.012 | 1533.2 | 801.555 | 265.312 | 253.558 |
| P-5 | C_6_H_5_NO_3_ | 138.168 | 77.791 | 55.951 | 13.371 | 7.409 | 14.378 |
| P-6 | C_6_H_6_O_3_ | 2067.516 | 1027.07 | 440.238 | 172.614 | 69.047 | 85.609 |
| P-7 | C_6_H_6_O_4_ | 110901.516 | 51384.617 | 16512.963 | 8528.604 | 2845.632 | 2749.65 |
| P-8 | C_6_H_4_O_2_ | 3333.3 | 1609.812 | 614.06 | 269.186 | 100.053 | 112.14 |
| P-9 | C_8_H_9_NO_5_ | 3265655.25 | 1320743.75 | 241918.906 | 213924.94 | 50099.484 | 29759.688 |

**Table S6.** Curve fitting parameters for Sn K-edge EXAFS for 5% Fe-SnS and 5% Fe-SnS/PMS.

| Sample | Path | CN | R(Å) | σ^2^ (Å^2^) | ΔE_0_ (eV) | R-factor |
| --- | --- | --- | --- | --- | --- | --- |
| 5% Fe-SnS | Sn-S | 1 | 2.51 | 0.005 | 7.53 | 0.036 |
|  | Sn-S | 2 | 2.62 | 0.005 |  |  |
|  | Sn-S | 2 | 3.27 | 0.01 |  |  |
|  | Sn-S | 1 | 3.55 | 0.01 |  |  |
| 5% Fe-SnS/PMS | Sn-S | 1 | 2.54 | 0.009 | 7 | 0.028 |
|  | Sn-S | 2 | 2.65 | 0.009 |  |  |
|  | Sn-S | 2 | 3.25 | 0.03 |  |  |
|  | Sn-S | 1 | 3.53 | 0.03 |  |  |
|  | Sn-O | 1.6 | 2.07 | 0.006 |  |  |

**Table S7**. Curve fitting parameters for Sn K-edge EXAFS for SnS and SnS/PMS.

| Sample | Path | CN | R(Å) | σ^2^ (Å^2^) | ΔE_0_ (eV) | R-factor |
| --- | --- | --- | --- | --- | --- | --- |
| SnS | Sn-S | 1 | 2.52 | 0.005 | 6.7 | 0.026 |
|  | Sn-S | 2 | 2.63 | 0.005 |  |  |
|  | Sn-S | 2 | 3.22 | 0.008 |  |  |
|  | Sn-S | 1 | 3.42 | 0.008 |  |  |
| SnS/PMS | Sn-S | 1 | 2.54 | 0.004 | 7 | 0.019 |
|  | Sn-S | 2 | 2.66 | 0.004 |  |  |
|  | Sn-S | 2 | 3.25 | 0.007 |  |  |
|  | Sn-S | 1 | 3.50 | 0.004 |  |  |
|  | Sn-O | 1.6 | 2.07 | 0.007 |  |  |

**Table S8.** Curve fitting parameters for Fe K-edge EXAFS for 5% Fe-SnS and 5% Fe-SnS/PMS.

| Sample | Path | CN | R(Å) | σ^2^ (Å^2^) | ΔE_0_ (eV) | R-factor |
| --- | --- | --- | --- | --- | --- | --- |
| 5% Fe-SnS | Fe-S | 6 | 2.30 | 0.016 | -0.59 | 0.016 |
| 5% Fe-SnS/PMS | Fe-S | 6 | 2.30 | 0.016 | -0.58 | 0.02 |

**Table S9.** Bader electron population and electron gain/loss relative to Z_VAL_ for Fe–SnS.

| Atom | N_Bader_ (e) | Z_VAL_ | Electron gain/loss, ΔN_e_ (e) |
| --- | --- | --- | --- |
| Sn 1 | 3.0627 | 4 | -0.9373 |
| Sn 2 | 3.0841 | 4 | -0.9159 |
| Sn 3 | 3.0601 | 4 | -0.9399 |
| Sn 4 | 3.0633 | 4 | -0.9367 |
| Sn 5 | 3.0459 | 4 | -0.9541 |
| Sn 6 | 3.0793 | 4 | -0.9207 |
| Sn 7 | 3.06 | 4 | -0.94 |
| Sn 8 | 3.0988 | 4 | -0.9012 |
| Sn 9 | 3.0806 | 4 | -0.9194 |
| Sn 10 | 3.0731 | 4 | -0.9269 |
| Sn 11 | 3.0821 | 4 | -0.9179 |
| Sn 12 | 3.0988 | 4 | -0.9012 |
| Sn 13 | 3.0848 | 4 | -0.9152 |
| Sn 14 | 3.0766 | 4 | -0.9234 |
| Sn 15 | 3.0818 | 4 | -0.9182 |
| Fe 16 | 7.1622 | 8 | -0.8378 |
| S 17 | 6.9084 | 6 | 0.9084 |
| S 18 | 6.9079 | 6 | 0.9079 |
| S 19 | 6.9194 | 6 | 0.9194 |
| S 20 | 6.9088 | 6 | 0.9088 |
| S 21 | 6.9128 | 6 | 0.9128 |
| S 22 | 6.908 | 6 | 0.908 |
| S 23 | 6.9198 | 6 | 0.9198 |
| S 24 | 6.918 | 6 | 0.918 |
| S 25 | 6.9308 | 6 | 0.9308 |
| S 26 | 6.9224 | 6 | 0.9224 |
| S 27 | 6.9238 | 6 | 0.9238 |
| S 28 | 6.9281 | 6 | 0.9281 |
| S 29 | 6.9238 | 6 | 0.9238 |
| S 30 | 6.9224 | 6 | 0.9224 |
| S 31 | 6.9238 | 6 | 0.9238 |
| S 32 | 6.9276 | 6 | 0.9276 |

**References**

1. N. Li, R. Li, X. Duan, et al., "Correlation of Active Sites to Generated Reactive Species and Degradation Routes of Organics in Peroxymonosulfate Activation by Co-Loaded Carbon," *Environmental Science & Technology* 55 (2021): 16163-16174, <https://doi.org/10.1021/acs.est.1c06244>.

2. G. Kresse, and J. Hafner, "Ab initio molecular dynamics for liquid metals," *Phys Rev B Condens Matter* 47 (1993): 558-561, <https://doi.org/10.1103/physrevb.47.558>.

3. G. K. J. Furthmu¨ller, "Efficient iterative schemes for ab initio total-energy calculations using a plane-wave basis set," *Physical Review B* 54 (1996): 11169-11185. https://doi.org/[10.1103/PhysRevB.54.11169](https://doi.org/10.1103/PhysRevB.54.11169).

4. K. B. John P. Perdew, * Matthias Ernzerhof, "Generalized Gradient Approximation Made Simple," *Physical Review Letters* 77 (1996): 3865-3868. https://doi.org/10.1103/PhysRevLett.77.3865.

5. P. E. Blochl, "Projector augmented-wave method," *Phys Rev B Condens Matter* 50 (1994): 17953-17979, <https://doi.org/10.1103/physrevb.50.17953>.

6. S. Grimme, J. Antony, S. Ehrlich, and H. Krieg, "A consistent and accurate ab initio parametrization of density functional dispersion correction (DFT-D) for the 94 elements H-Pu," *Journal of Chemical Physics* 132 (2010): 154104, <https://doi.org/10.1063/1.3382344>.

7. H. Khan, N. Mahmood, A. Zavabeti, et al., "Liquid metal-based synthesis of high performance monolayer SnS piezoelectric nanogenerators," *Nature Communications* 11 (2020): 3449, <https://doi.org/10.1038/s41467-020-17296-0>.

8. W. Tian, N. Li, D. Chen, et al., "Vibration-driven Reduction of CO2 to Acetate with 100% Selectivity by SnS Nanobelt Piezocatalysts," *Angewandte Chemie International Edition* 62 (2023): e202306964, <https://doi.org/10.1002/anie.202306964>.

9. T. J. Whittles, L. A. Burton, J. M. Skelton, A. Walsh, T. D. Veal, and V. R. Dhanak, "Band Alignments, Valence Bands, and Core Levels in the Tin Sulfides SnS, SnS2, and Sn2S3: Experiment and Theory," *Chemistry of Materials* 28 (2016): 3718-3726, <https://doi.org/10.1021/acs.chemmater.6b00397>.

10. Y. Li, Q. Wang, X. Zhang, et al., "Development of a heterogenous catalyst FeWO4/Cu2S as peroxymonosulfate activator for effective sulfachloropyridazine elimination," *Journal of Cleaner Production* 434 (2024), <https://doi.org/10.1016/j.jclepro.2023.140098>.

11. N. Li, Y. Wang, X. Cheng, et al., "Influences and mechanisms of phosphate ions onto persulfate activation and organic degradation in water treatment: A review," *Water Research* 222 (2022): 118896, <https://doi.org/10.1016/j.watres.2022.118896>.

12. Z. Wang, A. Xie, Z. Li, et al., "Selective oxidation of electron-rich pollutants in peroxymonosulfate-activated electro-Fenton system: The role of microenvironment-regulated cathode," *Water Research* 268 (2025): 122699, <https://doi.org/10.1016/j.watres.2024.122699>.

13. K. Qian, H. Chen, W. Li, Z. Ao, Y. N. Wu, and X. Guan, "Single-Atom Fe Catalyst Outperforms Its Homogeneous Counterpart for Activating Peroxymonosulfate to Achieve Effective Degradation of Organic Contaminants," *Environmental Science & Technology* 55 (2021): 7034-7043, <https://doi.org/10.1021/acs.est.0c08805>.

14. Y. Zhang, J. Niu, and J. Xu, "Fe(II)-promoted activation of peroxymonosulfate by molybdenum disulfide for effective degradation of acetaminophen," *Chemical Engineering Journal* 381 (2020): 122718, <https://doi.org/10.1016/j.cej.2019.122718>.

15. X. Liu, P. Xu, Q. Fu, et al., "Ferric ion promoted degradation of acetaminophen with zero − valent copper activated peroxymonosulfate process," *Chemical Engineering Journal* 426 (2021): 131679, <https://doi.org/10.1016/j.cej.2021.131679>.

16. S. Liu, P. Qi, S. Xu, et al., "Insights into nitrogen doped sulfur-existed carbonaceous material toward acetaminophen removal by peroxymonosulfate activation," *Journal of Environmental Chemical Engineering* 10 (2022): 107853, <https://doi.org/10.1016/j.jece.2022.107853>.

17. J. Ding, H. Nie, S. Wang, et al., "Transformation of acetaminophen in solution containing both peroxymonosulfate and chlorine: Performance, mechanism, and disinfection by-product formation," *Water Research* 189 (2021): 116605, <https://doi.org/10.1016/j.watres.2020.116605>.

18. F. Fanaei, G. Moussavi, V. Srivastava, and M. Sillanpää, "The enhanced catalytic potential of sulfur-doped MgO (S-MgO) nanoparticles in activation of peroxysulfates for advanced oxidation of acetaminophen," *Chemical Engineering Journal* 371 (2019): 404-413, <https://doi.org/10.1016/j.cej.2019.04.007>.

19. J. Li, J. Zou, S. Zhang, et al., "Sodium tetraborate simultaneously enhances the degradation of acetaminophen and reduces the formation potential of chlorinated by-products with heat-activated peroxymonosulfate oxidation," *Water Research* 224 (2022): 119095, <https://doi.org/10.1016/j.watres.2022.119095>.

20. H.-W. Lee, W.-G. Jeong, J.-G. Kim, and K. Baek, "MgO2-Mediated Alkaline Activation of Peroxymonosulfate (PMS) for Acetaminophen Degradation," *Korean Journal of Chemical Engineering* 43 (2026): 1391-1401, <https://doi.org/10.1007/s11814-026-00645-y>.

21. C. Tan, N. Gao, Y. Deng, et al., "Radical induced degradation of acetaminophen with Fe3O4 magnetic nanoparticles as heterogeneous activator of peroxymonosulfate," *Journal of Hazardous Materials* 276 (2014): 452-460, <https://doi.org/10.1016/j.jhazmat.2014.05.068>.

22. P. Sun, H. Liu, M. Feng, et al., "Dual nonradical degradation of acetaminophen by peroxymonosulfate activation with highly reusable and efficient N/S co-doped ordered mesoporous carbon," *Separation and Purification Technology* 268 (2021): 118697, <https://doi.org/10.1016/j.seppur.2021.118697>.

23. H. Meng, J. Zhou, Y. Zhang, et al., "Single-atom Co-N3 sites induce peroxymonosulfate activation for acetaminophen degradation via nearly 100 % internal electron transfer process," *Applied Catalysis B: Environment and Energy* 366 (2025): 125038, <https://doi.org/10.1016/j.apcatb.2025.125038>.

24. G. Liao, X. Qing, P. Xu, et al., "Synthesis of single atom cobalt dispersed on 2D carbon nanoplate and degradation of acetaminophen by peroxymonosulfate activation," *Chemical Engineering Journal* 427 (2022): 132027, <https://doi.org/10.1016/j.cej.2021.132027>.

25. M. Sun, W. Fang, Q. Liang, Y. Xing, L. Lin, and H. Luo, "Efficient acetaminophen degradation via peroxymonosulfate activation by Mn/N co-doped biochar: Performance, mechanism and approach," *Journal of Environmental Chemical Engineering* 12 (2024): 112647, <https://doi.org/10.1016/j.jece.2024.112647>.

26. S. Deng, Z. Shan, H. Zhang, et al., "Peroxymonosulfate activation by tin(IV) phosphate supported cobalt composite for acetaminophen degradation: Performance and mechanism," *Journal of Water Process Engineering* 61 (2024): 105343, <https://doi.org/10.1016/j.jwpe.2024.105343>.

27. S. Badiger, K. Kaur, and P. V. Nidheesh, "Iron-modified coconut shell biochar-activated peroxymonosulfate for acetaminophen degradation and microbial inactivation in sewage," *Environmental Science: Water Research & Technology* 11 (2025): 3161-3179, <https://doi.org/10.1039/d5ew00800j>.
